# Human Subcutaneous Adipose Transcriptomes Link Mitochondrial Transcription to Metabolic Heterogeneity in Obesity

**DOI:** 10.3390/genes17101199

**Published:** 2026-09-28

**Authors:** Shuang Bai, Meng Qi, Shuang Wu, Junzhu Wang, Qing Yang, Yajuan Cui, Siyuan Ma, Dongling Wang

**Affiliations:** 1 School of Kinesiology and Health, Capital University of Physical Education and Sports, Beijing 100191, China; baishuang@cupes.edu.cn (S.B.);; 2 Division of Sports Science and Physical Education, Tsinghua University, Beijing 100084, China; qimqmm@163.com; 3 College of Forestry, Beijing Forestry University, Beijing 100083, China; 4 Beijing Institute of Nutritional Resources Company Limited, Beijing 100069, China; 5 Central Laboratory, Peking University School and Hospital of Stomatology & National Center for Stomatology & National Clinical Research Center for Oral Diseases & National Engineering Research Center of Oral Biomaterials and Digital Medical Devices, Beijing 100081, China

**Keywords:** metabolically healthy obesity, metabolically unhealthy obesity, subcutaneous adipose tissue, transcriptomics, mitochondrial transcription, extracellular matrix, cell deconvolution

## Abstract

Background: Metabolically healthy obesity and metabolically unhealthy obesity may occupy different positions along a continuum of adipose-tissue dysfunction, but stable molecular distinctions remain uncertain. Methods: We integrated three public human subcutaneous-adipose-tissue transcriptomic datasets while preserving participant and platform independence. GSE244118 was used for discovery, GSE152991 was restricted to 14 participants not overlapping with the discovery cohort, and GSE55200 provided cross-platform supportive evidence. We performed differential-expression analysis, correlation-adjusted Reactome testing, direction-aware cross-dataset integration, discovery-defined module scoring, and adipose-tissue deconvolution. Results: Fifty genes met conventional thresholds for metabolically unhealthy versus metabolically healthy obesity in the discovery dataset, but none passed the prespecified fold-change-aware test or strict cross-dataset replication. Among 1570 shared Reactome pathways, 409 reached false-discovery rate < 0.05 in signed-Z integration and 387 had concordant directions across all datasets. Metabolically unhealthy obesity consistently showed lower mitochondrial translation, respiratory-chain, and energy-metabolism programs, accompanied by secondary increases in extracellular-matrix remodeling. A discovery-defined mitochondrial module was lower in metabolically unhealthy obesity in synthesis restricted to non-discovery datasets under both rank and z-mean scoring. The 14-person holdout alone did not establish a significant module difference, and the extracellular-matrix module synthesis was nonsignificant. Ordered cross-sectional patterns and composition sensitivity described tissue-level associations. Conclusions: Metabolic obesity heterogeneity was therefore more reproducible at pathway and module levels than at the single-gene level. Unresolved clinical confounding and bulk tissue composition limit functional and causal interpretation.

## 1. Introduction

Obesity is biologically heterogeneous. Some people with obesity retain comparatively favorable glucose, lipid, liver-fat, and insulin-sensitivity profiles and are described as having metabolically healthy obesity (MHO), whereas others develop metabolically unhealthy obesity (MUO) [1,2]. The distinction is clinically relevant but definition-dependent: meta-analyses and longitudinal cohorts show that MHO is not uniformly benign, may transition to metabolic dysfunction, and remains associated with elevated long-term cardiometabolic risk relative to metabolically healthy normal weight [3,4,5,6]. MHO and MUO should therefore be considered heterogeneous metabolic states rather than fixed diagnoses. Molecular features that distinguish these states could improve biological stratification, provided that cross-sectional signals are not overinterpreted as prognostic biomarkers.

Subcutaneous adipose tissue (SAT) is central to this heterogeneity because its capacity for relatively safe lipid storage can limit ectopic lipid delivery to the liver, skeletal muscle, and other organs. The adipose-expandability model, studies of adipocyte turnover, and contemporary adipogenesis research emphasize that tissue remodeling and the ability to recruit functional adipocytes are as important as total fat mass [7,8,9]. SAT is also an endocrine, vascular, immune, and structural organ that coordinates substrate handling, adipokine secretion, extracellular-matrix (ECM) turnover, and tissue repair [10,11,12]. When expansion becomes maladaptive, hypoxia, inflammation, fibrosis, impaired angiogenesis, and mitochondrial dysfunction can develop together and contribute to systemic insulin resistance [13,14,15].

Human studies support both mitochondrial and matrix-remodeling components of metabolic obesity. Detailed phenotyping has linked MHO to lower hepatic lipid content, greater whole-body insulin sensitivity, and distinct SAT transcriptional programs relative to MUO [16]. Other investigations have associated metabolic dysfunction with impaired adipose oxygenation [17], altered amino-acid and lipid metabolism [18], lower mitochondrial transcriptional activity [19,20,21], and increased fibrogenesis or ECM remodeling [22,23,24,25,26]. These observations suggest coordinated tissue programs rather than a single molecular switch.

Bulk SAT transcriptomes nevertheless average signals across mature adipocytes, progenitors, fibroblasts, endothelial and other vascular cells, and diverse immune populations. Single-cell and single-nucleus atlases reveal substantial depot-, disease-, and cell-state-specific heterogeneity in human adipose tissue [27,28,29,30]. Metabolic-health-focused single-nucleus studies and bulk deconvolution analyses further indicate that both cellular abundance and within-cell transcription can differ between MHO and MUO [31,32]. Consequently, a lower mitochondrial signal or a higher ECM signal in bulk tissue may reflect regulation within cells, altered cell composition, or both; computational deconvolution can test sensitivity to this issue but cannot resolve it completely [33].

Cross-study reproducibility presents a second challenge. MHO/MUO cohorts are generally small, phenotype definitions differ, and RNA-sequencing and microarray platforms have different dynamic ranges and annotation structures. Batch effects can alter expression levels and gene–gene correlation [34], while multi-laboratory and MicroArray Quality Control (MAQC) evaluations show that pathway-level biological themes may be more reproducible than individual measurements [35,36]. Methodological reviews of transcriptomic evidence synthesis therefore recommend separate within-dataset preprocessing, explicit assessment of study independence, and integration of comparable statistics rather than direct merging of heterogeneous expression matrices [37,38,39,40]. Longitudinal human studies also distinguish expression changes that reverse between dietary phases from obesity-associated changes retained after substantial weight loss [41,42]. This temporal heterogeneity makes it important to distinguish reproducibility across cohorts from persistence within individuals when interpreting cross-sectional SAT profiles.

Here, we integrated three public human SAT transcriptomic datasets while preserving participant and platform independence. GSE244118 served as the discovery and module-definition dataset; GSE152991 was restricted to participants who did not overlap with the discovery cohort; and GSE55200 provided cross-platform supportive evidence. We evaluated reproducibility hierarchically at the single-gene, Reactome-pathway, sample-level module, and estimated cell-composition levels. We hypothesized that metabolic obesity heterogeneity would be more reproducible as a coordinated mitochondrial and ECM program than as a stable individual-gene effect.

## 2. Materials and Methods

### 2.1. Study Design, Datasets, Phenotypes, and Participant Independence

This study was a secondary analysis of publicly available human SAT transcriptomic data. Dataset roles were defined to separate discovery from participant-independent and cross-platform evaluation. GSE244118 was used for discovery, three-group expression-pattern classification, and gene-module definition. GSE152991 was restricted to participants who did not overlap with GSE244118 and served as an RNA-sequencing holdout for the primary MUO–MHO contrast. GSE55200 served as cross-platform supportive evidence and, because it included all three phenotypes, as a second dataset for ordered cross-sectional patterns across metabolically healthy lean (MHL), MHO, and MUO groups.

The primary biological comparison was MUO minus MHO. Comparisons involving MHL were used to describe obesity-common changes and ordered cross-sectional phenotype patterns. Every expression matrix was preprocessed and modeled separately because platform and batch effects can distort direct cross-study expression comparisons and correlation structures [34,35]. No cross-platform expression matrix was constructed, and no classifier or clinical prediction model was trained. Exercise datasets, mouse datasets, cardiovascular event prediction, and external genetic analyses were outside the prespecified scope of this manuscript.

GSE244118 comprised bulk RNA-sequencing of abdominal SAT obtained from adults who underwent detailed cardiometabolic phenotyping [16]. The source study enrolled 15 MHL, 20 MHO, and 20 MUO participants; the available adipose RNA-sequencing matrix used here contained 15, 19, and 19 samples, respectively. MHO was defined by obesity together with normal fasting glucose, oral glucose tolerance, glycated hemoglobin, intrahepatic triglyceride content, serum triglycerides, and whole-body insulin sensitivity. MUO included impaired fasting glucose or glucose tolerance, hepatic steatosis, and impaired whole-body insulin sensitivity.

GSE152991 comprised abdominal SAT RNA-sequencing from 45 MHL, MHO, and MUO participants in a study of adipose oxygenation and insulin resistance [17]. We used the author-processed moderated log2 counts-per-million expression matrix. Participant and sample identifiers showed that 30 of the 45 participants also appeared in GSE244118. Excluding these participants left 15 samples. One MHL sample was not eligible for the primary MUO–MHO comparison, leaving 6 MHO and 8 MUO samples for the participant-nonoverlapping RNA-sequencing holdout. No MHL comparison from GSE152991 was included (Supplementary Table S1).

GSE55200 comprised Affymetrix Human Gene 2.1 ST profiles from periumbilical SAT obtained after an approximately 12 h fast [18]. The microarray subset contained 7 MHL, 8 MHO, and 8 MUO participants, with two men in each group (male/female counts, 2/5, 2/6, and 2/6). The original study classified participants using anthropometric and metabolic criteria, not adipose-expression clustering. Obesity was defined as body mass index (BMI) ≥ 28 kg/m^2^ in men or ≥24 kg/m^2^ in women, and metabolic health required at least three of five lipid and insulin-resistance criteria. We retained the original clinical labels and used this dataset as cross-platform supportive evidence because its platform and phenotype definitions differed from those of the discovery study.

Participant overlap was assessed using the source-study participant identifiers, sample titles, phenotype annotations, and accession metadata. A one-to-one overlap table was constructed before any replication analysis. Samples belonging to the 30 overlapping GSE152991 participants were excluded from all holdout tests. Technical samples and multiple records belonging to the same participant were not counted as independent replication. Clinical-linkage searches covered Gene Expression Omnibus (GEO) sample records, linked BioSample, Sequence Read Archive (SRA), and European Nucleotide Archive (ENA) records, and the original publications and scientific supplementary tables. Cell Data S1 contained 55 clinical records but lacked explicit identifiers linking them to the 53 SAT RNA-sequencing samples or identifying the two participants absent from that subset. Clinical records also could not be reliably linked to the 14-person GSE152991 holdout or individual GSE55200 expression samples. We therefore did not calculate unavailable subset-specific clinical summaries or test individual clinical associations from unlinked records. Supplementary Table S22 separates source-cohort information from characteristics reliably attributable to the analyzed subsets and lists the sources searched. Source-study phenotype thresholds and sampling protocols are also detailed there. The two RNA-sequencing studies obtained SAT during the basal clamp period following standardized inpatient meals; GSE55200 was sampled after an approximately 12 h overnight fast. These protocols do not establish control of individual habitual diets or recent weight change.

### 2.2. Expression Preprocessing and Differential-Expression Analysis

The GSE244118 gene-count matrix was analyzed in R using edgeR 4.4.0 [43,44]. Genes were retained with filterByExpr, using the metabolic group in the design, and library composition was normalized with the trimmed mean of M values (TMM; calcNormFactors (method = “TMM”)) [45]. TMM estimates scaling factors from the majority of genes after trimming extreme expression and fold-change values, thereby limiting composition bias between libraries. A no-intercept design matrix (~0 + metabolic_group) represented MHL, MHO, and MUO. Dispersions were estimated robustly, and quasi-likelihood negative-binomial models were fitted with glmQLFit (…, robust = TRUE). Contrasts were MHO–MHL, MUO–MHL, and MUO–MHO.

The conventional differentially expressed gene (DEG) definition required a two-sided Benjamini–Hochberg false discovery rate (FDR) below 0.05 and |log2 fold change| ≥ log2(1.5), approximately 0.585 [46]. Volcano plots colored only genes satisfying both criteria. This joint descriptive rule was distinguished from formal effect-threshold testing. Specifically, glmTreat tested the composite null that the true absolute fold change did not exceed log2(1.5), following the *t*-tests relative to a threshold (TREAT) framework [47]. A limma-voom model with precision and sample-quality weights was used as a sensitivity analysis [48,49]. Age, sex, BMI, and technical batch were not added because reliable sample-level covariate links were unavailable for the exact analyzed subsets. We did not infer clinical identities from row order, expression similarity, or full-cohort summaries.

Expression-pattern categories were defined from the three discovery contrasts using mutually exclusive logical rules implemented before pathway integration. Obesity-common genes changed in the same direction in both MHO–MHL and MUO–MHL; MHO-preserved/MUO-specific genes were unchanged in MHO–MHL but altered in MUO–MHL and MUO–MHO; MHO-protective candidates required a deviation in MHO opposite to the MUO-associated direction; and genes assigned to the descriptive progressive category showed an ordered cross-sectional MHL–MHO–MUO effect pattern. These classifications were descriptive and were not used as external validation outcomes.

The GSE152991 moderated log2 counts-per-million matrix and the author-processed GSE55200 microarray matrix were analyzed with limma 3.62.1 [49]. When multiple expression features mapped to one gene, the scripts retained a single gene-level value using the prespecified annotation and aggregation rules recorded in the dataset-specific code. Group effects were fitted using no-intercept design matrices, and contrasts were evaluated with empirical-Bayes moderation. The primary holdout/support comparison was MUO–MHO. The same DEG thresholds were used for descriptive comparability, although validation was evaluated through direction, effect concordance, full FDR, and candidate-set FDR. This layered evaluation was chosen because quantitative agreement is often weaker across expression platforms than agreement in broader biological themes [35]. Dataset-level expression quality-control displays are provided in Supplementary Figures S1, S5a, and S6a.

For candidate-gene replication, GSE244118 MUO–MHO candidates were mapped to each non-discovery dataset without ambiguous symbol assignments. We recorded the proportion with concordant effect direction, Spearman correlation of mapped log2 fold changes, nominal directional support, significance after correction within the candidate family, and strict replication based on full genome-wide multiplicity control. These evidence levels are reported separately.

### 2.3. Reactome Analysis and Direction-Aware Cross-Dataset Integration

Human Reactome Ensembl-to-pathway mappings were downloaded from Reactome and frozen with the project inputs; the corresponding file version and checksum are supplied in the code package [50]. Within each dataset, pathways containing 10–500 unique mapped genes were retained after mapping to the tested expression universe. The correlation-adjusted mean-rank (CAMERA) test evaluated the MUO–MHO contrast using the complete expression input and design within each dataset, rather than a selected DEG list [51]. This competitive test accounted for inter-gene correlation using the prespecified value of 0.01. Direction was taken from the CAMERA Up/Down output, and *p* values were adjusted with the Benjamini–Hochberg method within each dataset’s tested pathway family. Supplementary Table S21 reports each dataset’s mapped pathway size, direction, *p* value and FDR, together with the derived signed Z and integrated statistics.

The 1570 Reactome pathways tested in all three datasets formed the common meta-analysis universe. For pathway j in dataset i, the two-sided CAMERA *p* value p_ij_ was converted to an absolute normal score and multiplied by the CAMERA direction:

Z_ij_ = sign_ij_ Φ^−1^(1 − p_ij_/2).

The signed scores were combined with square-root contrast-sample-size weights. Here, n_i_ counts only MHO and MUO participants and equals 38, 14, and 16 for GSE244118, GSE152991, and GSE55200, respectively; MHL samples do not contribute to these weights:

Zmeta,j = (Σ_i_ √n_i_ Z_ij_)/√(Σ_i_ n_i_).

Two-sided meta-analysis *p* values were calculated from Zmeta and adjusted across the 1570 shared pathways. The inverse-normal procedure follows the weighted Z family of *p*-value combination methods, for which sample-size weighting can improve precision when studies test the same null hypothesis [52]. We additionally recorded direction concordance, the standard deviation of single-dataset signed Z statistics, and the minimum and maximum single-dataset *p* values. Because signed Z is a measure of combined statistical evidence rather than a standardized biological effect, conventional I^2^ was not calculated for this analysis. The analysis therefore tests cross-dataset directional evidence and does not estimate a pooled biological pathway effect size.

Fourteen representative Reactome pathways summarized the primary mitochondrial, ECM, branched-chain amino-acid, and RNA-processing findings. The set compressed nested or strongly overlapping terms and required meta-analysis FDR < 0.05 with concordant direction in all datasets. To assess selection stability, each dataset was held out in turn. Candidate pathways were re-derived from the other two datasets and then tested for expected direction, nominal significance, and strict FDR in the held-out dataset. Because all three datasets contributed to the overall pathway-discovery process, this procedure was interpreted as internal stability analysis rather than a prospectively specified external validation. We also repeated integration after omitting each dataset while retaining the fixed 1570-pathway universe, to distinguish evidence stability from changes in pathway selection.

This approach follows established guidance for pathway-level evidence synthesis across transcriptomic studies [39,40].

### 2.4. Module Definition, Scoring, and Cross-Dataset Evaluation

Modules were defined only from GSE244118 using canonical Reactome parent pathways. The mitochondrial-bioenergetics module combined R-HSA-5368287 (mitochondrial translation) and R-HSA-1428517 (aerobic respiration and respiratory electron transport). The ECM-remodeling module used R-HSA-1474244 (extracellular matrix organization). Parent pathways were required to satisfy FDR < 0.05 and the expected MUO–MHO direction in GSE244118. Genes shared across the two modules were removed to avoid mathematically coupled scores. The final modules contained 351 mitochondrial and 281 ECM genes. GSE152991 and GSE55200 did not contribute to gene selection. Frozen definitions, gene membership, and dataset-specific mapping coverage are provided in Supplementary Tables S2–S4.

For the primary rank score, genes were ranked within each sample using average ranks for ties. Ranks were divided by the number of genes in the processed expression matrix plus one. The score was the mean normalized rank of mapped module genes minus 0.5. For the z-mean sensitivity score, each gene was standardized across samples within a dataset and the mapped module-gene z scores were averaged. Each score required at least 10 mapped genes. Scores were never compared as raw values across platforms.

Within each dataset, the standardized module score was regressed on the metabolic group, and the MUO–MHO coefficient was evaluated with type 3 heteroskedasticity-consistent (HC3) standard errors using sandwich 3.1-1 and lmtest 0.9-40 [53,54]. Regression *p* values and 95% confidence intervals (CIs) used the t distribution with residual degrees of freedom. *p* values were adjusted across the two prespecified modules within each score/model family (Supplementary Tables S5 and S6). Separately, standardized mean differences were expressed as small-sample-corrected Hedges’ g with normal-approximation 95% CIs for effect-size synthesis [55].

The primary module synthesis included only GSE152991 and GSE55200, thereby excluding the module-definition dataset. Inverse-variance fixed-effect estimates were primary. DerSimonian–Laird random-effects models were sensitivity analyses [56]. We reported Cochran’s Q, I^2^, and τ^2^, while recognizing that between-study heterogeneity cannot be estimated reliably from only two non-discovery sample collections.

Ordered cross-sectional patterns were evaluated in GSE244118 and GSE55200, the two datasets containing MHL, MHO, and MUO. Phenotypes were encoded as MHL = 0, MHO = 1, and MUO = 2. Within-dataset standardized module scores were regressed on this ordinal variable with HC3 standard errors. Spearman correlation provided a rank-based sensitivity measure. The two module *p* values were adjusted within each dataset and scoring method. These cross-sectional trends were used to describe an ordered tissue-expression state and not longitudinal transition.

### 2.5. Adipose-Tissue Cell Deconvolution

Cell proportions were estimated with dtangle 2.0.9 [57] using a normalized human abdominal-SAT single-nucleus signature matrix and its optimized 6000 highly variable-gene list [32]. Genes were mapped to the signature using the frozen gene-information file and dataset-specific identifiers. dtangle used marker_method = “ratio” and a primary marker fraction of 0.10. Marker fractions of 0.05 and 0.20 were evaluated without changing the primary result. The two reference adipocyte subtypes were aggregated as total adipocytes, producing six broad cell families. Adipocytes, macrophages, and vascular cells were the three prespecified primary comparisons. Because bulk deconvolution accuracy depends on transformation, normalization, marker selection, and whether the reference includes the cell types present in the mixture, the estimates were treated as model-dependent proxies rather than measured fractions [33].

Estimated proportions were required to lie between zero and one and to sum to one within numerical tolerance. Because proportions are constrained to a simplex and changes in one component necessarily affect the others, they were analyzed after a centered log-ratio (CLR) transformation [58]:

clr(p_k_) = log[max(p_k_, 10^−6^)] − (1/K) Σ_l=1_^K^ log[max(p_l_, 10^−6^)].

Within each dataset, CLR values were modeled with limma and robust empirical-Bayes moderation. *p* values were adjusted across the three primary cell families. As an orthogonal analysis, cell-specific markers were selected from the reference signature and converted to sample-level rank scores without using the dtangle proportion estimates. HC3 regressions tested their MUO–MHO directions. Marker scores were interpreted as expression-based abundance proxies, not proportions or independent experimental validation. Cell-level tests, synthesis, and marker-score checks are reported in Supplementary Tables S7–S9; marker-fraction sensitivity and deconvolution quality checks are in Supplementary Tables S19a–c and S20a–c.

### 2.6. Cell-Composition Synthesis and Adjustment Sensitivity

MUO–MHO differences in each primary cell family were expressed as Hedges’ g and synthesized using fixed-effect and DerSimonian–Laird random-effects models. Random-effects estimates governed interpretation when heterogeneity was substantial, and FDR was controlled across the three cell families.

For the module-adjustment sensitivity analysis, CLR-transformed proportions from MHO and MUO samples underwent principal-component analysis within each dataset. The first two composition principal components (PC1 and PC2) were included in:

Module score ~ metabolic group + composition PC1 + composition PC2

and compared with the unadjusted model:

Module score ~ metabolic group

Both models used standardized rank-module scores and HC3 standard errors. Adjustment was performed in every dataset regardless of the unadjusted result. The analysis was not formulated as causal mediation because proportions were estimated, sample sizes were small, and cellular composition may be part of the phenotype rather than an external confounder. Regression intervals used the t distribution with residual degrees of freedom, consistent with the HC3 tests (Supplementary Table S10).

### 2.7. Multiple Testing, Software, and Reproducibility

All tests were two-sided. Benjamini–Hochberg adjustment was applied to full gene-level analyses, within-dataset CAMERA pathway families, the 1570-pathway signed-Z meta-analysis, the two-module families, and the three prespecified cell-composition families. Unless explicitly labeled as nominal or candidate-set evidence, significance denotes adjusted *p* < 0.05. Analyses were run in R 4.4.3 with edgeR 4.4.0, limma 3.62.1, dtangle 2.0.9, sandwich 3.1-1, lmtest 0.9-40, and data.table 1.18.4. The random seed was 20260730.

The complete workflow retained machine-readable inputs, intermediate results, software versions, and checksums in accordance with reproducible computational-research recommendations [59,60]. Supplementary Table S11 records design and interpretive risks; Supplementary Table S21 provides the complete shared-pathway results.

## 3. Results

### 3.1. Cohorts and Analytical Roles

The analysis comprised 90 separately analyzed SAT expression samples across three data collections (Figure 1; Table 1). GSE244118 contained abdominal SAT RNA-sequencing data from 15 metabolically healthy lean (MHL), 19 MHO, and 19 MUO participants and was assigned the discovery and module-definition role. The source study characterized MHO using normal fasting glucose, oral glucose tolerance, glycated hemoglobin, intrahepatic triglyceride, serum triglyceride, and whole-body insulin sensitivity, whereas MUO included prediabetes, hepatic steatosis, and insulin resistance [16].

GSE152991 contributed a 14-person participant-nonoverlapping RNA-sequencing holdout after exclusion of 30 overlapping participants and one remaining MHL sample outside the primary comparison. GSE55200 contributed 23 clinically classified microarray samples as cross-platform supportive evidence. Original full-cohort clinical summaries were not substituted for characteristics of these analyzed subsets. The known GSE55200 group-level sex counts do not identify sex for individual GEO samples.

Each expression matrix underwent dataset-specific preprocessing and statistical modeling. We did not merge expression values across platforms. Cross-dataset evidence was combined only at the levels of gene-effect direction, pathway statistics, or standardized module effects. This hierarchy separated discovery, participant-independent holdout testing, and cross-platform support while preventing participant overlap and direct matrix merging from inflating replication evidence.

### 3.2. Discovery-Stage Gene-Level Differences

In GSE244118, differential expression was assessed using an edgeR quasi-likelihood model, with threshold-defined differentially expressed genes (DEGs) required to satisfy both a Benjamini–Hochberg false-discovery rate (FDR) below 0.05 and an absolute log2 fold change of at least log2(1.5), approximately 0.585. MHO versus MHL yielded 1969 DEGs (1347 upregulated and 622 downregulated), and MUO versus MHL yielded 2958 DEGs (2070 upregulated and 888 downregulated) (Figure 2a,b,d). By contrast, MUO versus MHO produced 50 conventionally thresholded DEGs, comprising 45 upregulated and five downregulated genes (Figure 2c,d).

The interpretation changed when the fold-change threshold was incorporated into the null hypothesis. edgeR glmTreat directly tested whether each effect exceeded |log2 fold change| = log2(1.5), approximately 0.585, and confirmed 386 MHO–MHL and 1207 MUO–MHL genes, but no MUO–MHO gene. The 50 conventionally thresholded MUO–MHO DEGs therefore provided discovery-stage candidates rather than a robust set of large-effect differences. A limma-voom analysis was retained as model sensitivity evidence and was not used to replace the prespecified quasi-likelihood/TREAT interpretation.

We next classified genes using pre-specified three-group expression patterns. Obesity-common changes predominated, with 1719 genes changing in the same direction in both obese groups relative to MHL; 363 of these also satisfied the TREAT criterion in both obesity-versus-lean contrasts. In comparison, seven genes showed an MHO-preserved/MUO-specific pattern, no gene met the MHO-protective candidate definition, and 37 showed progressive dysregulation across MHL, MHO, and MUO (Figure 2e,f). These categories are descriptive features of the discovery dataset rather than validated biomarkers. Collectively, the discovery-stage landscape was dominated by transcriptional changes shared across obesity phenotypes, whereas large-effect MHO–MUO differences were sparse and were not confirmed by the prespecified TREAT analysis. Full pattern counts and complementary sample-level and group-mean heatmaps are shown in Supplementary Table S12 and Figures S2 and S3.

### 3.3. Limited Replication of Candidate Genes

The participant-independent GSE152991 holdout contained no gene satisfying the genome-wide DEG definition for MUO versus MHO. Of the discovery candidates, 34 had unambiguous mappings and could be tested in the holdout. Fourteen showed the same direction of effect, but effect-size concordance was weak (Spearman ρ = 0.049, *p* = 0.783; Figure 3a). Two genes had at least nominal directional support, one of which remained significant after candidate-set correction, and none met the strict replication definition requiring compatible direction and full genome-wide evidence (Figure 3b). Individual candidate-level findings were therefore not interpreted as genome-wide replication. Further holdout candidate results are provided in Supplementary Table S13 and Figure S5b.

In GSE55200, 41 discovery candidates could be mapped across platforms. Thirty-three had concordant effect directions, which exceeded the 50% sign expectation (exact binomial *p* = 5.61 × 10^−5^), but the magnitudes of the effects remained uncorrelated (Spearman ρ = 0.040, *p* = 0.806; Figure 3c). Three candidates had nominal directional support, but none showed candidate-set or strict genome-wide replication. The excess of concordant signs and the absence of effect-size correlation address different aspects of reproducibility: a common weak directional tendency does not establish stable quantitative gene effects. These findings provide cross-platform directional support, but not quantitative replication of individual-gene effects. Supplementary Table S15 and Figure S6b provide the corresponding candidate-level support results.

Thus, discovery-stage candidate genes showed limited and inconsistent effect-size replication in both the participant-independent holdout and the cross-platform supportive dataset. This did not imply the absence of MHO–MUO biology; rather, it indicated that individual genes were not the most stable unit of evidence in these sample collections.

### 3.4. Cross-Dataset Convergence of Mitochondrial and ECM Pathways

We therefore evaluated coordinated biological programs using CAMERA competitive gene-set testing of the MUO–MHO contrast. The test used the full expression input within each dataset and accounted for inter-gene correlation using a fixed value of 0.01; it did not depend on selecting threshold-defined DEGs. Pathway direction was taken from the CAMERA Up/Down output.

A total of 1570 Reactome pathways were tested in all three sample collections. For each pathway, the two-sided CAMERA *p* value was transformed to a signed Z statistic and combined across datasets with square-root contrast-sample-size weights. Four hundred and nine pathways met meta-analysis FDR < 0.05, of which 387 had the same direction in all three datasets (Figure 4a). Signed Z quantified combined directional statistical evidence and was not treated as a biological effect size; conventional effect-size heterogeneity measures were therefore not applied to these pathway Z statistics.

The most coherent downregulated programs in MUO involved mitochondrial translation, mitochondrial ribosome-associated quality control, respiratory electron transport, aerobic respiration, cristae formation, the tricarboxylic-acid cycle, and branched-chain amino-acid catabolism. Fibronectin matrix formation and ECM proteoglycan pathways were consistently upregulated. A representative frozen set of 14 Reactome pathways summarized these related findings without treating nested pathway terms as independent mechanisms (Figure 4b). All 14 had meta-analysis FDR < 0.05 and concordant directions across the three datasets. In three leave-one-dataset-out cycles, pathways defined from the remaining two datasets retained their expected direction in the held-out dataset, and 14 reached the strict held-out criterion in each cycle.

The 14-pathway set was derived from the available cross-cohort evidence, and the leave-one-dataset-out analysis is therefore an internal selection-stability assessment rather than a prospective external validation. Within that boundary, the results show that the reproducible distinction between MHO and MUO was concentrated in coordinated mitochondrial and matrix-remodeling programs rather than stable individual genes. Complete cross-dataset pathway summaries and selection-stability results are provided in Supplementary Tables S14 and S16–S18, and all shared pathways in  Table S21. Supplementary Figures S4, S5c, and S6c retain exploratory pathway displays using their explicitly labeled thresholds; these displays do not change the primary FDR < 0.05 criterion. Supplementary Table S23 reports all 1570 pathways for each fixed-universe leave-one-dataset-out analysis.

### 3.5. Module Evidence and Ordered Cross-Sectional Patterns

To convert the pathway findings into sample-level measurements without using validation datasets for gene selection, we defined two modules exclusively in GSE244118. The mitochondrial-bioenergetics module combined genes in the canonical mitochondrial-translation and aerobic-respiration/respiratory-electron-transport parent pathways, and the ECM-remodeling module used the canonical ECM-organization parent pathway. Parent pathways were required to have the expected direction and FDR < 0.05 in the discovery dataset. After removing cross-module overlaps, the mitochondrial and ECM modules contained 351 and 281 genes, respectively. Dataset-specific mapped coverage ranged from approximately 88.9% to 100%.

The primary module score was the mean within-sample percentile rank of mapped module genes minus 0.5. A mean of within-dataset gene z scores provided a sensitivity score. Scores were calculated separately within each dataset. In a synthesis restricted to non-discovery datasets of GSE152991 and GSE55200, the mitochondrial module was lower in MUO under both scoring methods. The fixed-effect Hedges’ g was −1.004 (95% confidence interval [CI], −1.735 to −0.274; *p* = 0.00702; FDR = 0.0140) for the rank score and −0.955 (95% CI, −1.679 to −0.230; *p* = 0.00985; FDR = 0.0197) for the z-mean score (Figure 5a; Table 2). Estimated I^2^ was 0% for both analyses, although two non-discovery sample collections are insufficient to establish an absence of heterogeneity. In the 14-person GSE152991 holdout alone, the HC3 module tests did not reach FDR < 0.05 (rank FDR = 0.218; z-mean FDR = 0.161; Supplementary Table S5).

The ECM module had the expected positive direction but weaker evidence in the non-discovery synthesis. For the rank score, Hedges’ g was 0.707 (95% CI, −0.0005 to 1.415; *p* = 0.0501; FDR = 0.0501). For the z-mean score, Hedges’ g was 0.508 (95% CI, −0.189 to 1.206; *p* = 0.153; FDR = 0.153). Neither result met the prespecified 0.05 threshold. Thus, pathway-level ECM convergence did not establish a significant broad ECM module effect in the non-discovery synthesis.

Both datasets containing all three phenotypes showed ordered cross-sectional module patterns (Figure 5b). In GSE244118, the standardized rank-score trend from MHL to MHO to MUO was −0.631 for the mitochondrial module (FDR = 4.45 × 10^−4^; Spearman ρ = −0.463) and 0.928 for the ECM module (FDR = 2.41 × 10^−8^; ρ = 0.801). In GSE55200, the corresponding trends were −0.982 for the mitochondrial module (FDR = 1.55 × 10^−5^; ρ = −0.803) and 1.030 for the ECM module (FDR = 2.64 × 10^−6^; ρ = 0.784). These cross-sectional patterns placed MHO between MHL and MUO at the level of the bulk SAT transcriptome; they do not demonstrate that individual participants will progress from MHO to MUO. Supplementary Tables S5 and S6 report the complete module and ordered-pattern tests. The exploratory gene-z-score summaries in Supplementary Figure S6d are distinct from the frozen modules evaluated here.

### 3.6. Cell-Composition Sensitivity

We estimated SAT cell composition using dtangle, an abdominal-adipose single-nucleus reference signature, and a primary marker fraction of 10%; marker fractions of 5% and 20% were sensitivity analyses. Two adipocyte subtypes were aggregated before the prespecified comparisons. Estimated proportions passed range and closure checks, were transformed with a centered log ratio (CLR), and were tested for adipocytes, macrophages, and vascular cells. Rank-based cell-specific marker scores, computed independently of the dtangle proportions, provided an orthogonal direction check.

Across datasets, estimated adipocyte abundance was lower in MUO than in MHO (random-effects Hedges’ g = −0.601; 95% CI, −1.073 to −0.129; FDR = 0.0378; I^2^ = 0%; Figure 6a). Macrophage estimates were directionally higher but heterogeneous (g = 0.395; 95% CI, −0.583 to 1.374; FDR = 0.621; I^2^ = 74.0%). Vascular-cell estimates were directionally lower and similarly heterogeneous (g = −0.292; 95% CI, −1.451 to 0.867; FDR = 0.621; I^2^ = 81.2%). Thus, only the lower estimated adipocyte abundance had stable cross-dataset support; the macrophage and vascular-cell results could not be generalized across cohorts.

We next compared module models before and after adjustment for the first two principal components of CLR-transformed cellular composition (Figure 6b). In GSE244118, the standardized MUO–MHO coefficient decreased from 1.104 to 0.575 for the ECM module and from −0.404 to −0.149 for the mitochondrial module. In the GSE152991 holdout, the ECM estimate changed from 0.447 to 0.629 and the mitochondrial estimate from −0.884 to −0.911. In GSE55200, the ECM estimate changed from 0.951 to 0.940 and the mitochondrial estimate from −1.028 to −1.035, although uncertainty widened after adjustment. The discovery mitochondrial contrast was not significant before or after adjustment (*p* = 0.230 and 0.717), rather than changing from significant to nonsignificant. The corresponding ECM *p* values were 0.000360 and 0.0668 (Supplementary Table S10).

These analyses indicate that the discovery-dataset module contrasts were sensitive to estimated tissue composition, whereas the directions in the two non-discovery sample collections were not uniformly eliminated by the same adjustment. Because the cell proportions were computationally estimated and may themselves lie on the biological pathway between metabolic state and bulk transcription, these models are sensitivity analyses rather than mediation analyses. Bulk data cannot distinguish a lower abundance of adipocytes from altered expression within adipocytes or other resident cell types.

## 4. Discussion

### 4.1. Mitochondrial Transcriptional Programs Show Cross-Dataset Convergence

This cross-cohort analysis indicates that MHO–MUO differences in bulk SAT were more consistent at the pathway level than as individual-gene effects. Discovery-stage candidates failed formal fold-change-threshold testing and replicated poorly across the two non-discovery collections. In contrast, separately analyzed RNA-sequencing and microarray datasets converged on lower mitochondrial translation, respiratory-chain, and energy-metabolism programs in MUO. The discovery-defined mitochondrial module had a negative effect in the synthesis restricted to non-discovery datasets under both scoring strategies, although the 14-person RNA-sequencing holdout did not individually establish a significant module difference. These findings support mitochondrial transcriptional associations, not definitive validation of a biomarker or a direct measure of mitochondrial function.

The breadth of the mitochondrial result is biologically informative. The convergent terms spanned mitochondrial translation, ribosome quality control, electron transport, aerobic respiration, cristae formation, and the tricarboxylic-acid cycle rather than one respiratory-chain gene or complex. Adipocyte fuel storage, oxidation, and endocrine function depend on coordinated mitochondrial and nutrient-sensing programs, and maladaptation of these programs accompanies adipose dysfunction and insulin resistance [20,21]. Human SAT studies similarly report lower mitochondrial transcriptional activity in obesity and associations between adipose metabolic programs, whole-body insulin sensitivity, and metabolic health [16,19]. The present analysis extends those observations by showing that the direction of the signal was concordant across independent sample collections and distinct expression platforms.

The mitochondrial module should not be interpreted as a direct assay of organelle function. Lower RNA abundance does not establish lower respiratory flux, adenosine triphosphate production, mitochondrial mass, or substrate oxidation. Bulk expression also reflects the relative RNA contribution of resident cell populations. Functional respirometry, mitochondrial proteomics, and cell-type-resolved transcriptomics in the same clinically phenotyped participants are needed to distinguish reduced mitochondrial activity within adipocytes from altered adipocyte abundance or state.

### 4.2. ECM Remodeling and Cell Composition Provide Secondary Context

ECM-related pathways were increased in MUO, in agreement with evidence linking adipose fibrosis, impaired tissue expandability, and insulin resistance [22,23,24,25,26]. ECM remodeling can modify mechanical signaling, oxygen diffusion, vascular support, adipogenesis, and stromal-cell behavior [10,13]. However, the broad ECM module did not reach the prespecified significance threshold in the non-discovery synthesis under either scoring method. Competitive pathway tests and broad sample-level module tests evaluate different hypotheses, so pathway significance cannot substitute for module evidence. ECM remodeling is therefore a secondary, context-dependent transcriptional feature rather than a result with support equivalent to that of the mitochondrial module synthesis.

Estimated adipocyte abundance was lower in MUO, whereas macrophage and vascular-cell estimates were heterogeneous. Adjustment for composition principal components attenuated both module contrasts in GSE244118 but did not uniformly remove their direction in the non-discovery datasets. Human adipose atlases demonstrate extensive diversity among adipocytes, progenitors, fibroblasts, vascular cells, and immune populations [27,28,29,30]; metabolic-health-focused work also shows that abundance and cell state vary between MHO and MUO [31]. The bulk modules should therefore not be interpreted as purely cell-intrinsic.

Conversely, composition is not necessarily a nuisance factor. Reduced mature-adipocyte representation, impaired adipogenesis, structural-cell expansion, or immune recruitment may be part of the biological process linking tissue remodeling to metabolic dysfunction. Deconvolution depends on reference composition, marker specificity, expression scale, and completeness of the cell catalog [32,33]. The adjusted models are consequently sensitivity analyses for composition dependence, not causal mediation tests.

### 4.3. Temporal Plasticity and Persistence of Adipose Transcription

The ordered MHL–MHO–MUO cross-sectional patterns provide a sample-level representation of metabolic obesity heterogeneity. In both three-group datasets, MHO occupied an intermediate bulk-expression state characterized by more preserved mitochondrial programs and less pronounced ECM remodeling than MUO. This pattern is compatible with contemporary views of MHO as heterogeneous and potentially transitional [1,2,5], but it does not imply that every individual with MHO will progress to MUO or that the module score is a longitudinal predictor.

Intervention studies illustrate both phase-dependent and more persistent transcriptional changes. Exercise can remodel adipose tissue, although responses depend on tissue and intervention context [61,62]. In the Diet, Obesity and Genes (DiOGenes) study, human SAT expression of *SCD*, *FADS1* and *FADS2* decreased during eight weeks of calorie restriction and increased during the subsequent 26-week weight-maintenance phase [41]. These lipid-metabolism transcripts exemplify potentially reversible responses to changing energy balance, whereas *LEP* remained downregulated through maintenance [41]. At a longer timescale, human single-nucleus data showed that reduced expression of *IGF1*, *DUSP1*, *GPX3* and *GLUL* in subcutaneous adipocytes was retained two years after bariatric surgery [42]. The latter study supports persistence of selected human transcriptional abnormalities; its mechanistic evidence for epigenetic memory came from mice.

These observations suggest that some nutrient-responsive metabolic transcripts may be transitory, whereas selected obesity-associated changes can outlast substantial weight loss. Persistence within an observation window does not establish permanence or irreversibility, because body weight and environmental conditions may remain altered. In addition, the examples do not justify classifying all metabolic genes as transient or all ECM genes as persistent. Our mitochondrial and ECM signals could contain both dynamic and retained components. Their cross-cohort concordance does not identify their duration within individuals, and ECM transcription is not a direct measure of persistent fibrosis. Repeated SAT sampling with documented diet, weight trajectories and matched clinical measurements is required to distinguish these possibilities.

### 4.4. Strengths, Limitations, and Future Directions

The limited single-gene replication is scientifically informative. Human SAT cohorts are small and heterogeneous, platform-specific measurement error affects quantitative agreement, and direct matrix merging can confound biological and technical variation [34,35,36]. The analytical design addressed these constraints by auditing participant overlap, preprocessing each dataset separately, testing pathways with inter-gene-correlation adjustment, defining modules only in the discovery dataset, and synthesizing only comparable pathway statistics or standardized module effects. The signed-Z procedure is a meta-analytic method for combining directional evidence, not a pooled expression analysis and not an estimate of a biological effect size [39,40,52]. Leave-one-dataset-out analysis assessed internal stability without presenting the 14 representative pathways as prospectively validated.

Additional strengths include two module-scoring strategies, synthesis restricted to non-discovery datasets, adipose-specific deconvolution, marker-score checks, composition-adjusted sensitivity analyses, and retained analysis code [59,60]. Important limitations remain. The RNA-sequencing holdout contained only 14 participants, metabolic-health definitions differed across studies, and the data were cross-sectional bulk SAT measurements. Although public source tables contain clinical measurements, explicit links to the analyzed expression subsets were unavailable. Age, sex, BMI and other clinical differences may therefore contribute to the observed associations; cross-dataset concordance and cell-composition adjustment do not remove this residual confounding. The known group-level sex counts in GSE55200 cannot establish comparability in the other datasets. Estimated cell proportions and only two non-discovery collections further limit interpretation. Finally, the 14-pathway set was selected using the available datasets, making leave-one-dataset-out results evidence of internal stability rather than prospective external confirmation. Sex is particularly relevant because a human SAT study reported higher expression of mitochondrial-function genes in women than in men [63]; unavailable individual sex links prevent evaluating its contribution in our retained samples.

Future work should evaluate the frozen modules in a completely independent, clinically defined MHO/MUO SAT cohort; relate them to continuous insulin sensitivity, hepatic fat, glucose, and lipid phenotypes; and combine bulk profiles with single-nucleus, spatial, proteomic, and functional mitochondrial measurements. These steps are needed before the modules can be considered predictive, causal, or suitable for individual treatment selection.

## 5. Conclusions

Separate analyses of human SAT transcriptomes identified mitochondrial transcriptional programs associated with metabolic obesity heterogeneity. Pathway-level convergence and mitochondrial module synthesis restricted to non-discovery datasets provided stronger support than individual-gene replication, whereas the broad ECM module synthesis did not reach the prespecified significance threshold. Ordered cross-sectional expression patterns and composition sensitivity describe tissue-level associations, but do not establish longitudinal progression, cell-intrinsic mitochondrial dysfunction, or the temporal persistence of the identified transcriptional patterns. Small samples and unresolved clinical confounding preclude causal, diagnostic, or treatment-selection claims.

## Figures and Tables

**Figure 1:**
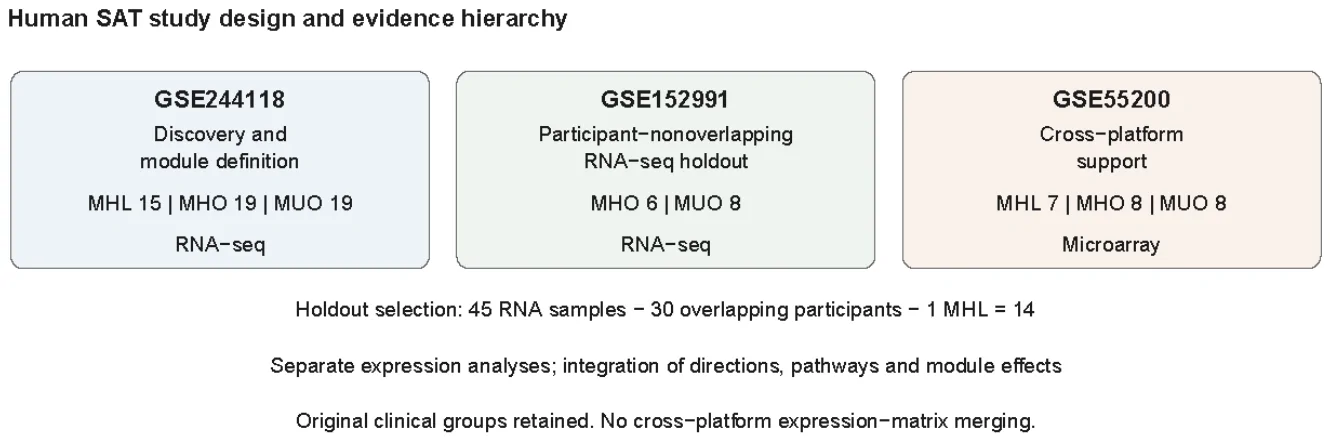
GSE244118 was used for discovery and module definition. Of 45 GSE152991 participants, 30 overlapped with GSE244118. After their exclusion, one remaining MHL sample was outside the primary comparison, leaving 6 MHO and 8 MUO participants in the participant-nonoverlapping RNA-sequencing holdout. GSE55200 provided cross-platform supportive evidence using the original clinically defined groups. Its microarray subset comprised 7 MHL, 8 MHO, and 8 MUO participants, including two men per group. Each expression matrix was analyzed separately; matrices from different platforms were not merged. MHL, metabolically healthy lean; MHO, metabolically healthy obesity; MUO, metabolically unhealthy obesity.

**Figure 2:**
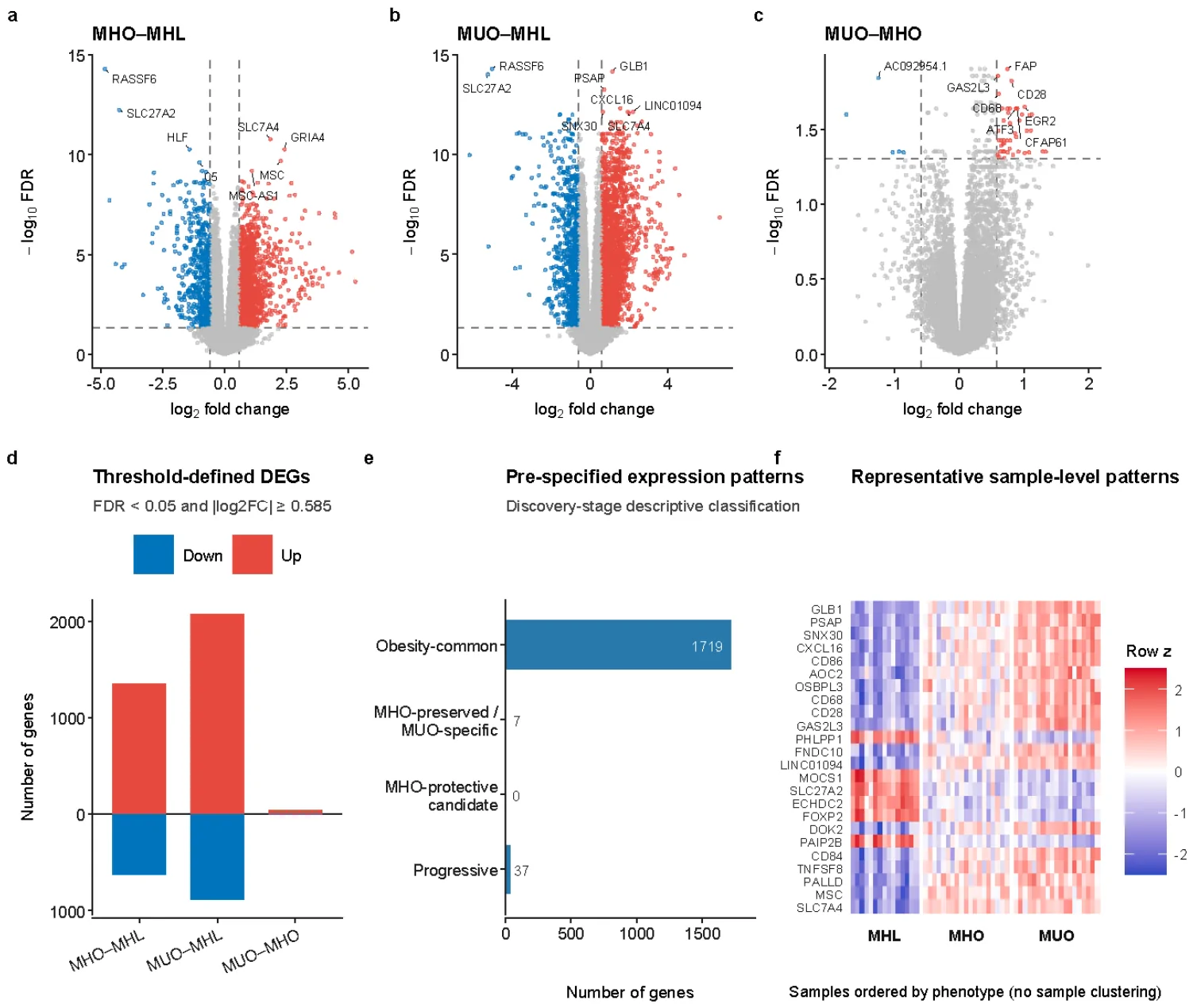
(**a**–**c**) Volcano plots for MHO versus MHL, MUO versus MHL, and MUO versus MHO, respectively. Differential expression was estimated using edgeR quasi-likelihood models. Red and blue denote genes satisfying both Benjamini–Hochberg false-discovery rate (FDR) < 0.05 and |log2 fold change| ≥ log2(1.5), approximately 0.585; gray denotes genes not satisfying the joint criterion. (**d**) Numbers of upregulated and downregulated genes in the three contrasts. (**e**) Numbers of genes in prespecified three-group expression-pattern categories. (**f**) Representative sample-level standardized-expression heatmap. Samples are ordered by phenotype (MHL, MHO, MUO) and are not clustered; the display is descriptive and does not define a classifier. Conventional threshold results are reported separately from glmTreat, which tested whether the absolute effect exceeded the prespecified fold-change threshold.

**Figure 3:**
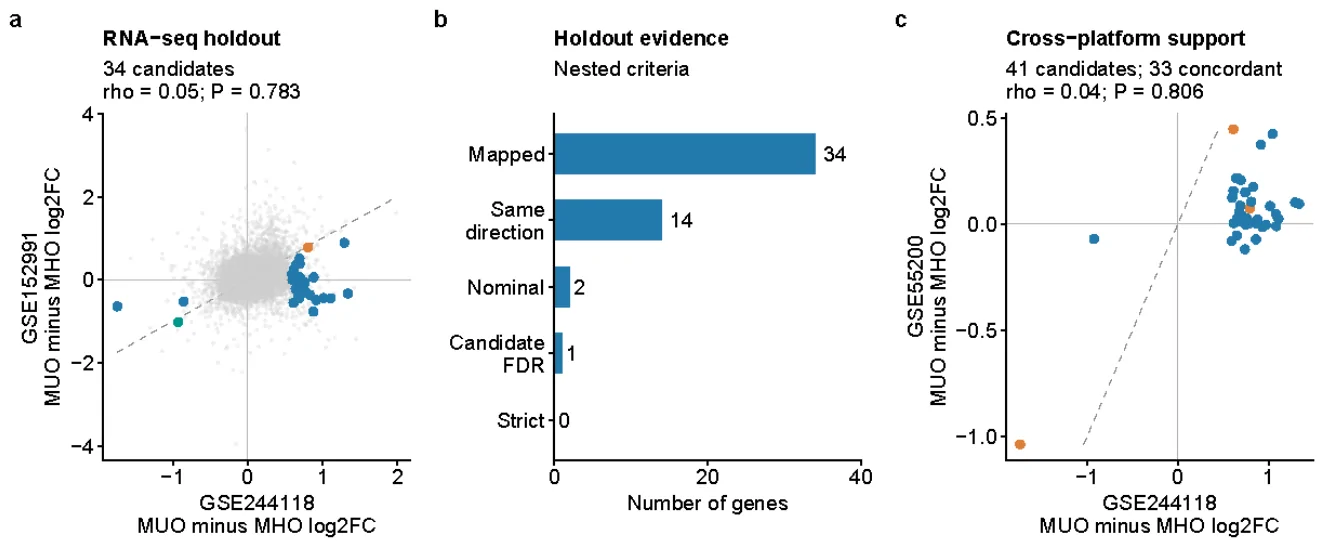
(**a**) Discovery and participant-nonoverlapping GSE152991 effect estimates. Gray points show all 14,816 mapped genes; colored points identify 34 discovery candidates. (**b**) Nested holdout evidence levels comprise 34 mapped candidates, 14 directionally concordant, 2 with nominal directional support, 1 passing candidate-set FDR correction, and 0 meeting the strict genome-wide replication definition. (**c**) All 41 mapped discovery candidates in the cross-platform supportive GSE55200 dataset. In (**a**,**c**), blue denotes candidates without directional statistical support, orange nominal directional support only, and green candidate-set FDR support. Dashed lines indicate equal effects; axis scales differ. Spearman correlations and two-sided *p* values use all mapped discovery candidates in each dataset. GSE55200 retained the original clinical labels. Directional concordance does not imply stable effect-size replication.

**Figure 4:**
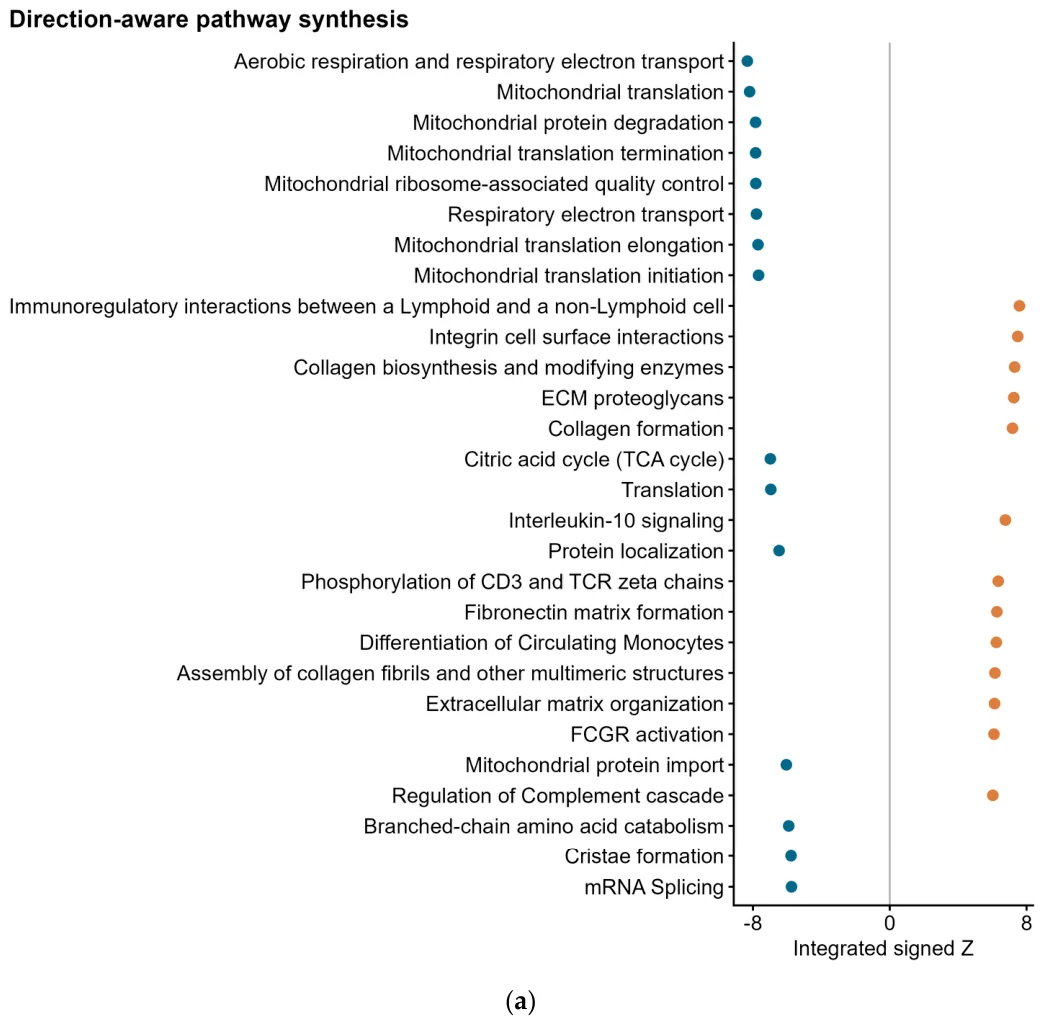

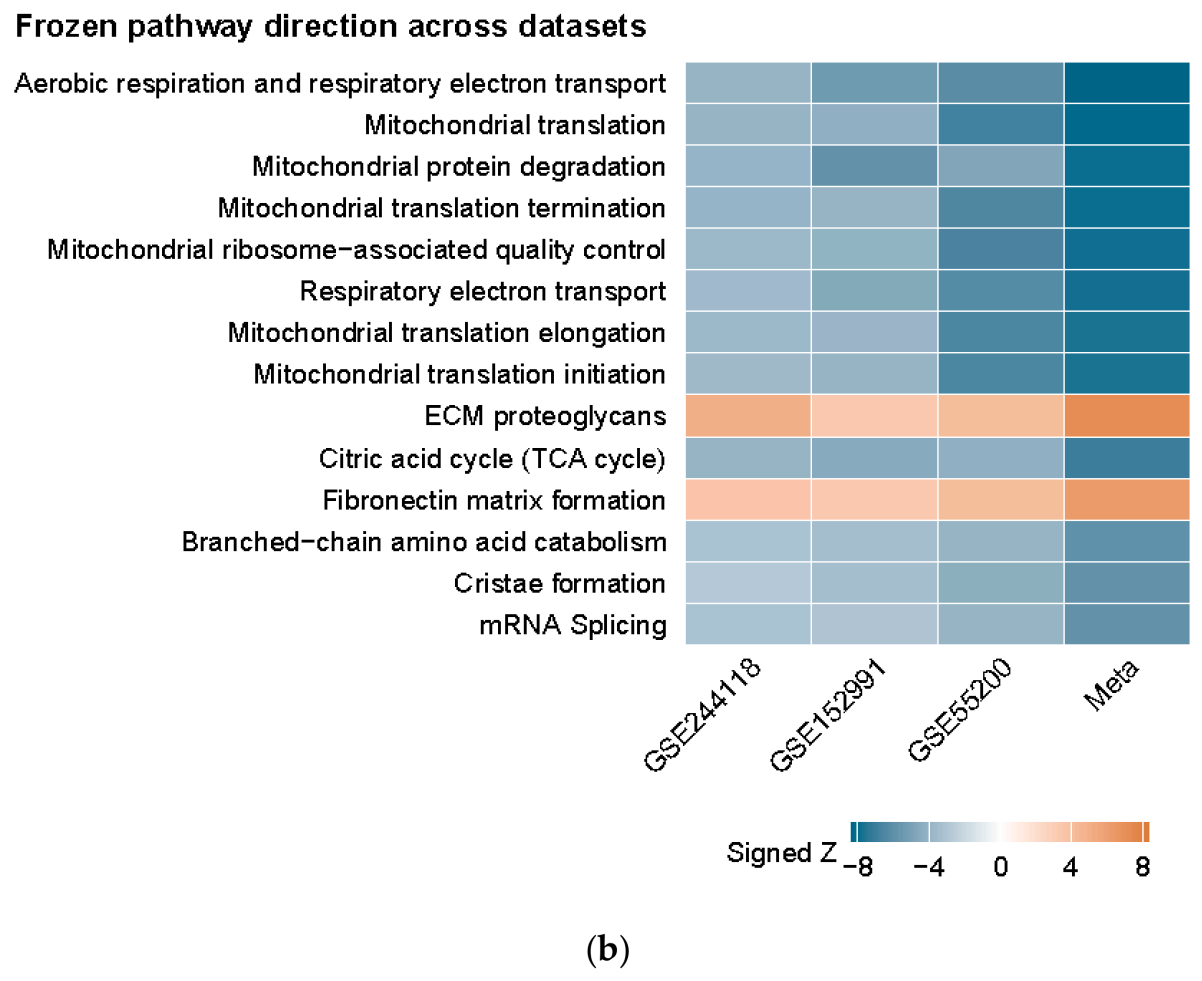
(**a**) Integrated signed Z for 28 displayed Reactome pathways, comprising the original top-25 selection and the frozen 14-pathway set after removing overlap. The complete tested universe comprised 1570 pathways (Supplementary Table S21). CAMERA *p* values were converted to signed Z and combined using square-root contrast-sample-size weights (√38, √14 and √16); Benjamini–Hochberg correction used the complete shared universe. Blue denotes lower expression in MUO and orange higher expression. (**b**) Signed Z for the 14 frozen pathways in each dataset and their integrated result (Meta). These biologically overlapping pathways were selected from the available datasets and are not 14 independent mechanisms or prospective validation targets. Signed Z measures directional statistical evidence, not a biological effect size.

**Figure 5:**
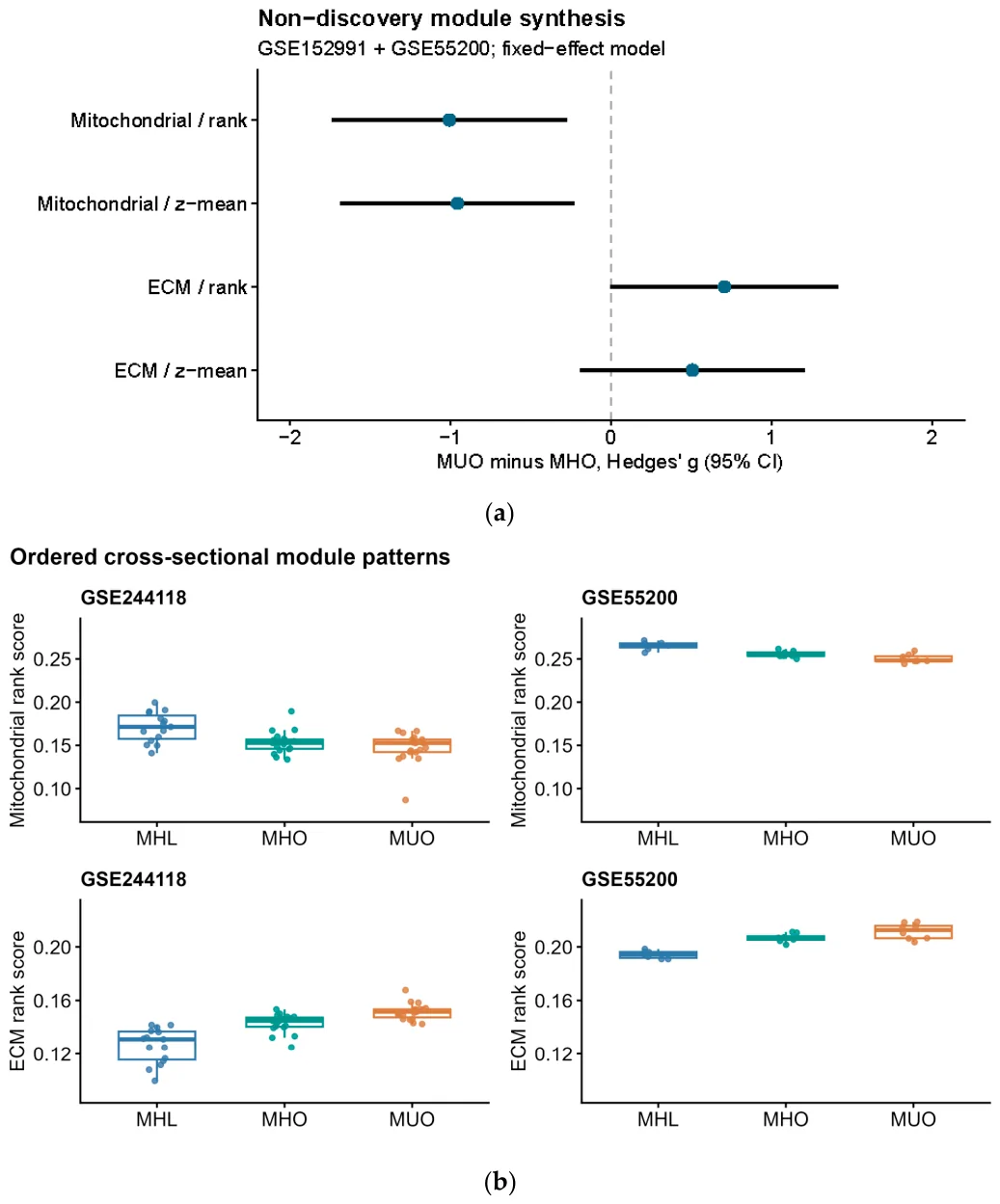
(**a**) Hedges’ g for MUO-minus-MHO mitochondrial-bioenergetics and extracellular-matrix (ECM)-remodeling module scores in the nonoverlapping GSE152991 subset and GSE55200. Rank scoring was the primary method; the mean within-dataset gene z score was a sensitivity analysis. Fixed-effect synthesis was primary, with random-effects results reported as sensitivity analyses. GSE55200 provides cross-platform support; the synthesis does not imply that each dataset separately establishes a significant module effect. (**b**) Sample-level rank-based mitochondrial-bioenergetics and ECM-remodeling scores across MHL, MHO, and MUO in GSE244118 and GSE55200. Boxes show medians and interquartile ranges; whiskers extend to 1.5 times the interquartile range, and points denote samples. Ordered cross-sectional patterns must not be interpreted as longitudinal progression.

**Figure 6:**
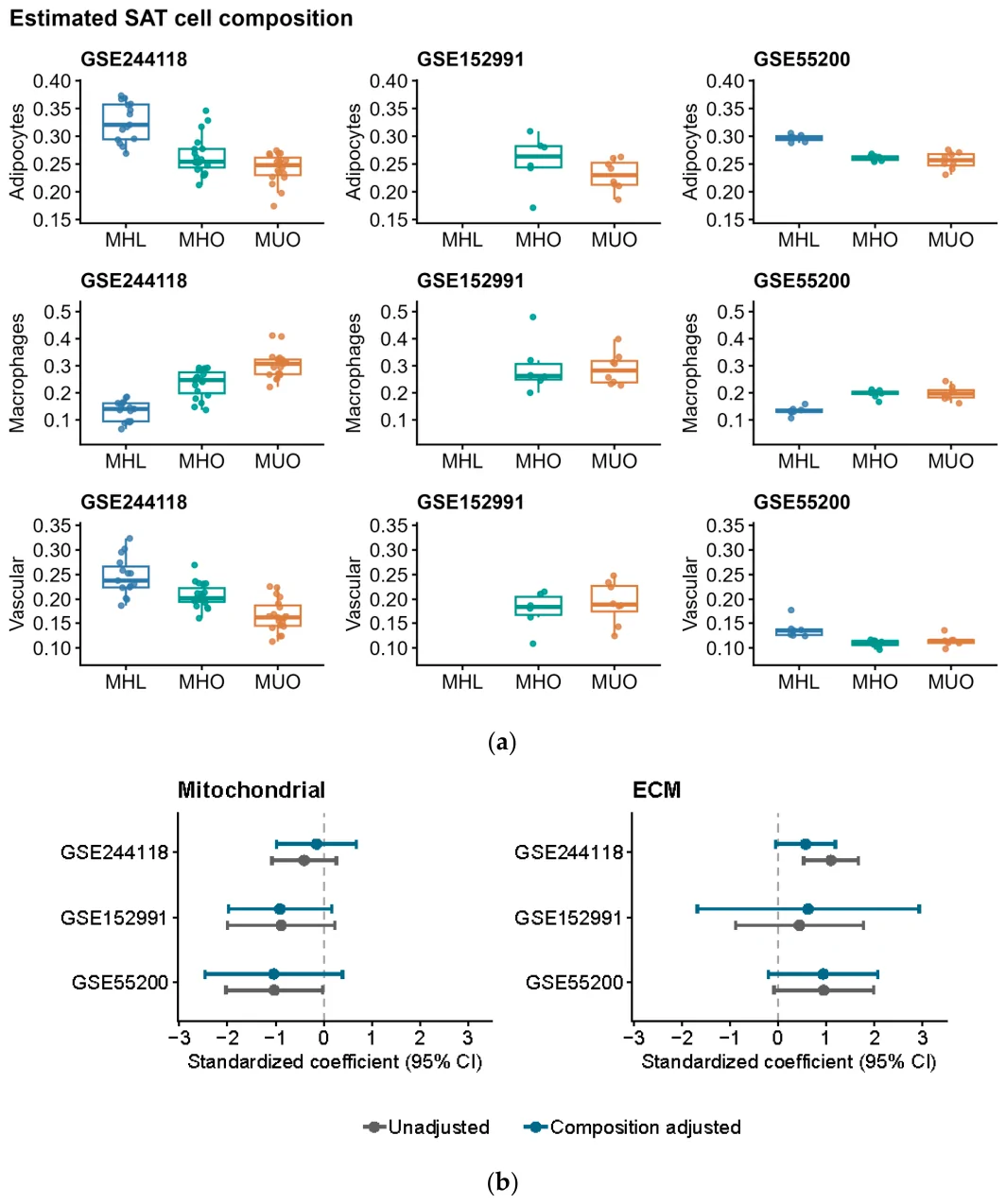
(**a**) Estimated adipocyte, macrophage, and vascular-cell proportions in GSE244118, the nonoverlapping GSE152991 subset, and GSE55200. Proportions were inferred using dtangle with a human abdominal-adipose reference and a 10% marker fraction; adipocyte subtypes were aggregated before prespecified cross-dataset tests. Boxes show medians and interquartile ranges; whiskers extend to 1.5 times the interquartile range, and points denote samples. GSE152991 contains no participant-independent MHL group. (**b**) Standardized MUO-minus-MHO module coefficients and HC3 t-based 95% confidence intervals using residual degrees of freedom before and after adjustment for the first two principal components of centered-log-ratio-transformed cell-composition estimates. Because the proportions are estimated and cell composition may lie on the biological pathway, this analysis assesses sensitivity to composition and does not establish mediation.

**Table 1:** Public datasets, phenotype definitions, overlap, and analytical roles.

Dataset	Tissue and Platform	Analyzed Groups and Sample Size	Phenotype Information Used in the Present Analysis	Participant Overlap	Analytical Role
GSE244118	Abdominal SAT; Illumina NovaSeq 6000 bulk RNA sequencing	MHL 15; MHO 19; MUO 19; total 53. *	MHO: normal fasting glucose, glucose tolerance, glycated hemoglobin (HbA1c), intrahepatic triglyceride, serum triglycerides, and whole-body insulin sensitivity. MUO: prediabetes, hepatic steatosis, and impaired insulin sensitivity	Reference dataset for overlap audit	Discovery, expression-pattern definition, and module definition
GSE152991	Abdominal SAT; author-processed moderated log2 counts per million from RNA sequencing	MHO 6; MUO 8; total 14. *	Source MHL/MHO/MUO classification; present analysis restricted to nonoverlapping obese participants	Of 45 samples, 30 overlapping participants were excluded; one remaining MHL sample was outside the primary contrast	Participant-nonoverlapping RNA sequencing holdout for MUO minus MHO
GSE55200	Periumbilical SAT; Affymetrix Human Gene 2.1 ST microarray	MHL 7; MHO 8; MUO 8; total 23. *	Original anthropometric and metabolic criteria [18]; obesity BMI ≥ 28 in men or ≥24 kg/m^2^ in women; metabolic health requires at least three of five lipid/insulin-resistance criteria	No identified overlap with the two RNA-sequencing datasets	Cross-platform supportive dataset and ordered cross-sectional pattern analysis

* Individual clinical records could not be reliably linked to the analyzed expression subsets. Subset age, BMI summaries and sex counts for the RNA subsets are therefore unavailable; full-cohort summaries were not substituted. GSE55200 male/female counts were 2/5, 2/6 and 2/6 for MHL, MHO and MUO, respectively [18], but individual GEO sample sex was not identifiable. Classification thresholds are not observed subset means. Source-cohort and subset information is separated in Supplementary Table S22.

**Table 2:** Synthesis of discovery-defined module scores restricted to non-discovery datasets.

Score	Module	Model	k	Hedges’ g, MUO Minus MHO	95% CI	*p*	FDR Within Score/Model
Rank	Mitochondrial bioenergetics	Fixed effect	2	−1.004	−1.735 to −0.274	0.00702	0.0140
Rank	ECM remodeling	Fixed effect	2	0.707	−0.0005 to 1.415	0.0501	0.0501
Z mean	Mitochondrial bioenergetics	Fixed effect	2	−0.955	−1.679 to −0.230	0.00985	0.0197
Z mean	ECM remodeling	Fixed effect	2	0.508	−0.189 to 1.206	0.153	0.153

## Data Availability

The transcriptomic datasets analyzed in this study are publicly available from the National Center for Biotechnology Information Gene Expression Omnibus (https://ncbi.nlm.nih.gov/ accessed on 15 August 2026) under accession numbers GSE244118, GSE152991, and GSE55200. GSE244118 served as the discovery dataset; the analysis of GSE152991 was restricted to participants not overlapping with the discovery cohort; and GSE55200 provided cross-platform supportive evidence. The analysis code and source data underlying the main figures are publicly available at https://github.com/sololuoo/genes-human-adipose-code (accessed on 15 August 2026).

## Supplementary Materials

The following supporting information can be downloaded at: https://www.mdpi.com/article/10.3390/genes17101199/s1, Figure S1: GSE244118 quality control; Figure S2: Discovery expression patterns; Figure S3. Discovery group-mean expression; Figure S4. Exploratory discovery Reactome pathways; Figure S5a. GSE152991 quality control; Figure S5b. GSE152991 gene-level concordance; Figure S5c. Exploratory GSE152991 pathway concordance; Figure S6a. GSE55200 quality control; Figure S6b. GSE55200 candidate-gene support; Figure S6c. GSE55200 pathway support; Figure S6d. Exploratory GSE55200 pathway scores; Table S1: Participant-level overlap audit between GSE244118 and GSE152991; Table S2: Discovery-only module-freezing record; Table S3: Frozen mitochondrial-bioenergetics and ECM-remodeling module membership; Table S4: Module coverage by dataset and scoring method; Table S5: Dataset-specific MUO-MHO module tests; Table S6: Ordered MHL-MHO-MUO module-trend tests; Table S7: Dataset-specific cell-composition tests; Table S8: Cross-dataset synthesis of cell-composition effects; Table S9: Orthogonal cell-type marker-score tests; Table S10: Cell-composition-adjusted module sensitivity analysis; Table S11: Statistical-design and interpretation-risk audit; Table S12: Discovery differential-expression summary; Table S13: GSE152991 non-overlapping gene-replication summary; Table S14: GSE152991 pathway-replication summary; Table S15: GSE55200 candidate-gene supportive analysis; Table S16: GSE55200 pathway supportive analysis; Table S17: Direction-aware meta-analysis of the frozen 14 Reactome pathways; Table S18: Leave-one-dataset-out Reactome stability summary; Table S19a: Marker-fraction sensitivity analysis for adipose deconvolution—GSE244118; Table S19b: Marker-fraction sensitivity analysis for adipose deconvolution—GSE152991; Table S19c: Marker-fraction sensitivity analysis for adipose deconvolution—GSE55200; Table S20a: Deconvolution overlap, bounds and closure quality-control checks—GSE244118; Table S20b: Deconvolution overlap, bounds and closure quality-control checks—GSE152991; Table S20c: Deconvolution overlap, bounds and closure quality-control checks—GSE55200; Table S21: Direction-aware meta-analysis of all 1570 shared Reactome pathways; Table S22: Clinical source information and analyzed-subset availability [16,17,18]; Table S23: Leave-one-dataset-out pathway synthesis.

## References

[1] Smith G.I., Mittendorfer B., Klein S. (2019). Metabolically healthy obesity: Facts and fantasies. J. Clin. Investig..

[2] Schulze M.B., Stefan N. (2024). Metabolically healthy obesity: From epidemiology and mechanisms to clinical implications. Nat. Rev. Endocrinol..

[3] Kramer C.K., Zinman B., Retnakaran R. (2013). Are Metabolically Healthy Overweight and Obesity Benign Conditions?. Ann. Intern. Med..

[4] Eckel N., Meidtner K., Kalle-Uhlmann T., Stefan N., Schulze M.B. (2016). Metabolically healthy obesity and cardiovascular events: A systematic review and meta-analysis. Eur. J. Prev. Cardiol..

[5] Mongraw-Chaffin M., Foster M.C., Anderson C.A.M., Burke G.L., Haq N., Kalyani R.R., Ouyang P., Sibley C.T., Tracy R., Woodward M. (2018). Metabolically Healthy Obesity, Transition to Metabolic Syndrome, and Cardiovascular Risk. J. Am. Coll. Cardiol..

[6] Stefan N., Häring H.U., Hu F.B., Schulze M.B. (2013). Metabolically healthy obesity: Epidemiology, mechanisms, and clinical implications. Lancet Diabetes Endocrinol..

[7] Virtue S., Vidal-Puig A. (2010). Adipose tissue expandability, lipotoxicity and the Metabolic Syndrome—An allostatic perspective. Biochim. Biophys. Acta (BBA)—Mol. Cell Biol. Lipids.

[8] Spalding K.L., Arner E., Westermark P.O., Bernard S., Buchholz B.A., Bergmann O., Blomqvist L., Hoffstedt J., Näslund E., Britton T. (2008). Dynamics of fat cell turnover in humans. Nature.

[9] Ghaben A.L., Scherer P.E. (2019). Adipogenesis and metabolic health. Nat. Rev. Mol. Cell Biol..

[10] Sun K., Kusminski C.M., Scherer P.E. (2011). Adipose tissue remodeling and obesity. J. Clin. Investig..

[11] Rosen E.D., Spiegelman B.M. (2014). What We Talk About When We Talk About Fat. Cell.

[12] Scheja L., Heeren J. (2019). The endocrine function of adipose tissues in health and cardiometabolic disease. Nat. Rev. Endocrinol..

[13] Crewe C., An Y.A., Scherer P.E. (2017). The ominous triad of adipose tissue dysfunction: Inflammation, fibrosis, and impaired angiogenesis. J. Clin. Investig..

[14] Reilly S.M., Saltiel A.R. (2017). Adapting to obesity with adipose tissue inflammation. Nat. Rev. Endocrinol..

[15] Kusminski C.M., Bickel P.E., Scherer P.E. (2016). Targeting adipose tissue in the treatment of obesity-associated diabetes. Nat. Rev. Drug Discov..

[16] Petersen M.C., Smith G.I., Palacios H.H., Farabi S.S., Yoshino M., Yoshino J., Cho K., Davila-Roman V.G., Shankaran M., Barve R.A. (2024). Cardiometabolic characteristics of people with metabolically healthy and unhealthy obesity. Cell Metab..

[17] Cifarelli V., Beeman S.C., Smith G.I., Yoshino J., Morozov D., Beals J.W., Kayser B.D., Watrous J.D., Jain M., Patterson B.W. (2020). Decreased adipose tissue oxygenation associates with insulin resistance in individuals with obesity. J. Clin. Investig..

[18] Badoud F., Lam K.P., DiBattista A., Perreault M., Zulyniak M.A., Cattrysse B., Stephenson S., Britz-McKibbin P., Mutch D.M. (2014). Serum and Adipose Tissue Amino Acid Homeostasis in the Metabolically Healthy Obese. J. Proteome Res..

[19] Heinonen S., Muniandy M., Buzkova J., Mardinoglu A., Rodríguez A., Frühbeck G., Hakkarainen A., Lundbom J., Lundbom N., Kaprio J. (2017). Mitochondria-related transcriptional signature is downregulated in adipocytes in obesity: A study of young healthy MZ twins. Diabetologia.

[20] Kusminski C.M., Scherer P.E. (2012). Mitochondrial dysfunction in white adipose tissue. Trends Endocrinol. Metab..

[21] Chouchani E.T., Kajimura S. (2019). Metabolic adaptation and maladaptation in adipose tissue. Nat. Metab..

[22] Beals J.W., Smith G.I., Shankaran M., Fuchs A., Schweitzer G.G., Yoshino J., Field T., Matthews M., Nyangau E., Morozov D. (2021). Increased Adipose Tissue Fibrogenesis, Not Impaired Expandability, Is Associated with Nonalcoholic Fatty Liver Disease. Hepatology.

[23] Divoux A., Tordjman J., Lacasa D., Veyrie N., Hugol D., Aissat A., Basdevant A., Guerre-Millo M., Poitou C., Zucker J.D. (2010). Fibrosis in Human Adipose Tissue: Composition, Distribution, and Link with Lipid Metabolism and Fat Mass Loss. Diabetes.

[24] Henegar C., Tordjman J., Achard V., Lacasa D., Cremer I., Guerre-Millo M., Poitou C., Basdevant A., Stich V., Viguerie N. (2008). Adipose tissue transcriptomic signature highlights the pathological relevance of extracellular matrix in human obesity. Genome Biol..

[25] Marcelin G., Ferreira A., Liu Y., Atlan M., Aron-Wisnewsky J., Pelloux V., Botbol Y., Ambrosini M., Fradet M., Rouault C. (2017). A PDGFRα-Mediated Switch toward CD9high Adipocyte Progenitors Controls Obesity-Induced Adipose Tissue Fibrosis. Cell Metab..

[26] Pellegrinelli V., Rodriguez-Cuenca S., Rouault C., Figueroa-Juarez E., Schilbert H., Virtue S., Moreno-Navarrete J.M., Bidault G., Vázquez-Borrego M.C., Dias A.R. (2022). Dysregulation of macrophage PEPD in obesity determines adipose tissue fibro-inflammation and insulin resistance. Nat. Metab..

[27] Emont M.P., Jacobs C., Essene A.L., Pant D., Tenen D., Colleluori G., Di Vincenzo A., Jørgensen A.M., Dashti H., Stefek A. (2022). A single-cell atlas of human and mouse white adipose tissue. Nature.

[28] Vijay J., Gauthier M.F., Biswell R.L., Louiselle D.A., Johnston J.J., Cheung W.A., Belden B., Pramatarova A., Biertho L., Gibson M. (2020). Single-cell analysis of human adipose tissue identifies depot- and disease-specific cell types. Nat. Metab..

[29] Massier L., Jalkanen J., Elmastas M., Zhong J., Wang T., Nono Nankam P.A., Frendo-Cumbo S., Bäckdahl J., Subramanian N., Sekine T. (2023). An integrated single cell and spatial transcriptomic map of human white adipose tissue. Nat. Commun..

[30] Merrick D., Sakers A., Irgebay Z., Okada C., Calvert C., Morley M.P., Percec I., Seale P. (2019). Identification of a mesenchymal progenitor cell hierarchy in adipose tissue. Science.

[31] Reinisch I., Ghosh A., Noé F., Sun W., Dong H., Leary P., Dietrich A., Hoffmann A., Blüher M., Wolfrum C. (2025). Unveiling adipose populations linked to metabolic health in obesity. Cell Metab..

[32] Ahn C., Divoux A., Zhou M., Seldin M.M., Sparks L.M., Whytock K.L. (2025). Optimized RNA sequencing deconvolution illustrates the impact of obesity and weight loss on cell composition of human adipose tissue. Obesity.

[33] Avila Cobos F., Alquicira-Hernandez J., Powell J.E., Mestdagh P., De Preter K. (2020). Benchmarking of cell type deconvolution pipelines for transcriptomics data. Nat. Commun..

[34] Leek J.T., Scharpf R.B., Bravo H.C., Simcha D., Langmead B., Johnson W.E., Geman D., Baggerly K., Irizarry R.A. (2010). Tackling the widespread and critical impact of batch effects in high-throughput data. Nat. Rev. Genet..

[35] Members of the Toxicogenomics Research Consortium (2005). Standardizing global gene expression analysis between laboratories and across platforms. Nat. Methods.

[36] Shi L., Reid L.H., Jones W.D., Shippy R., Warrington J.A., Baker S.C., Collins P.J., de Longueville F., Kawasaki E.S., Lee K.Y. (2006). The MicroArray Quality Control (MAQC) project shows inter- and intraplatform reproducibility of gene expression measurements. Nat. Biotechnol..

[37] Ramasamy A., Mondry A., Holmes C.C., Altman D.G. (2008). Key Issues in Conducting a Meta-Analysis of Gene Expression Microarray Datasets. PLoS Med..

[38] Toro-Domínguez D., Villatoro-García J.A., Martorell-Marugán J., Román-Montoya Y., Alarcón-Riquelme M.E., Carmona-Sáez P. (2021). A survey of gene expression meta-analysis: Methods and applications. Brief. Bioinform..

[39] Shen K., Tseng G.C. (2010). Meta-analysis for pathway enrichment analysis when combining multiple genomic studies. Bioinformatics.

[40] Tseng G.C., Ghosh D., Feingold E. (2012). Comprehensive literature review and statistical considerations for microarray meta-analysis. Nucleic Acids Res..

[41] Viguerie N., Montastier E., Maoret J.-J., Roussel B., Combes M., Valle C., Villa-Vialaneix N., Iacovoni J.S., Martinez J.A., Holst C. (2012). Determinants of Human Adipose Tissue Gene Expression: Impact of Diet, Sex, Metabolic Status, and Cis Genetic Regulation. PLoS Genet..

[42] Hinte L.C., Castellano-Castillo D., Ghosh A., Melrose K., Gasser E., Noé F., Massier L., Dong H., Sun W., Hoffmann A. (2024). Adipose tissue retains an epigenetic memory of obesity after weight loss. Nature.

[43] Robinson M.D., McCarthy D.J., Smyth G.K. (2010). edgeR: A Bioconductor package for differential expression analysis of digital gene expression data. Bioinformatics.

[44] Chen Y., Lun A.T.L., Smyth G.K. (2016). From reads to genes to pathways: Differential expression analysis of RNA-Seq experiments using Rsubread and the edgeR quasi-likelihood pipeline. F1000Res.

[45] Robinson M.D., Oshlack A. (2010). A scaling normalization method for differential expression analysis of RNA-seq data. Genome Biol..

[46] Benjamini Y., Hochberg Y. (1995). Controlling the False Discovery Rate: A Practical and Powerful Approach to Multiple Testing. J. R. Stat. Soc. Ser. B Stat. Methodol..

[47] McCarthy D.J., Smyth G.K. (2009). Testing significance relative to a fold-change threshold is a TREAT. Bioinformatics.

[48] Law C.W., Chen Y., Shi W., Smyth G.K. (2014). voom: Precision weights unlock linear model analysis tools for RNA-seq read counts. Genome Biol..

[49] Ritchie M.E., Phipson B., Wu D., Hu Y., Law C.W., Shi W., Smyth G.K. (2015). limma powers differential expression analyses for RNA-sequencing and microarray studies. Nucleic Acids Res..

[50] Milacic M., Beavers D., Conley P., Gong C., Gillespie M., Griss J., Haw R., Jassal B., Matthews L., May B. (2024). The Reactome Pathway Knowledgebase 2024. Nucleic Acids Res..

[51] Wu D., Smyth G.K. (2012). Camera: A competitive gene set test accounting for inter-gene correlation. Nucleic Acids Res..

[52] Whitlock M.C. (2005). Combining probability from independent tests: The weighted Z-method is superior to Fisher’s approach. J. Evol. Biol..

[53] Zeileis A. (2004). Econometric Computing with HC and HAC Covariance Matrix Estimators. J. Stat. Softw..

[54] Long J.S., Ervin L.H. (2000). Using Heteroscedasticity Consistent Standard Errors in the Linear Regression Model. Am. Stat..

[55] Hedges L.V., Olkin I. (1985). Statistical Methods for Meta-Analysis.

[56] DerSimonian R., Laird N. (1986). Meta-analysis in clinical trials. Control. Clin. Trials.

[57] Hunt G.J., Freytag S., Bahlo M., Gagnon-Bartsch J.A. (2019). dtangle: Accurate and robust cell type deconvolution. Bioinformatics.

[58] Aitchison J. (1982). The Statistical Analysis of Compositional Data. J. R. Stat. Soc. Ser. B Stat. Methodol..

[59] Sandve G.K., Nekrutenko A., Taylor J., Hovig E. (2013). Ten Simple Rules for Reproducible Computational Research. PLoS Comput. Biol..

[60] Wratten L., Wilm A., Göke J. (2021). Reproducible, scalable, and shareable analysis pipelines with bioinformatics workflow managers. Nat. Methods.

[61] Lehnig A.C., Stanford K.I. (2018). Exercise-induced adaptations to white and brown adipose tissue. J. Exp. Biol..

[62] Stanford K.I., Middelbeek R.J.W., Townsend K.L., Lee M.Y., Takahashi H., So K., Hitchcox K.M., Markan K.R., Hellbach K., Hirshman M.F. (2015). A Novel Role for Subcutaneous Adipose Tissue in Exercise-Induced Improvements in Glucose Homeostasis. Diabetes.

[63] Nookaew I., Svensson P.-A., Jacobson P., Jernås M., Taube M., Larsson I., Andersson-Assarsson J.C., Sjöström L., Froguel P., Walley A. (2013). Adipose Tissue Resting Energy Expenditure and Expression of Genes Involved in Mitochondrial Function Are Higher in Women than in Men. J. Clin. Endocrinol. Metab..

