# Supplementary material for: Human Subcutaneous Adipose Transcriptomes Link Mitochondrial Transcription to Metabolic Heterogeneity in Obesity
Source: Genes (Basel). 2026 Sep 28;17(10):1199. doi: 10.3390/genes17101199 (PMC13704403; doi:10.3390/genes17101199)
Supplement: Supplementary file 1 [file genes-17-01199-s001.zip › genes-4536375_Supplementary_Figures.pdf]

# Supplementary Information

Human Subcutaneous Adipose Transcriptomes Link Mitochondrial Transcription to Metabolic Heterogeneity in Obesity

## Figures S1-S6

Figures S5a-c and S6a-d are stored on separate pages. Original vector panels and exact numerical source data accompany this document. Main-text figures are not duplicated.

**Figure S1** — GSE244118 quality control (page 2)

**Figure S2** — Discovery expression patterns (page 3)

**Figure S3** — Discovery group-mean expression (page 4)

**Figure S4** — Exploratory discovery Reactome pathways (page 5)

**Figure S5a** — GSE152991 quality control (page 6)

**Figure S5b** — GSE152991 gene-level concordance (page 7)

**Figure S5c** — Exploratory GSE152991 pathway concordance (page 8)

**Figure S6a** — GSE55200 quality control (page 9)

**Figure S6b** — GSE55200 candidate-gene support (page 10)

**Figure S6c** — GSE55200 pathway support (page 11)

**Figure S6d** — Exploratory GSE55200 pathway scores (page 12)

## Interpretive notes

GSE152991 uses only participant-nonoverlapping obese samples. GSE55200 provides cross-platform supportive evidence using source-defined clinical groups. Differences in phenotype definitions, platform and sample size limit comparability. Historical FDR < 0.10 displays are identified in their captions and are distinct from formal FDR < 0.05 synthesis.

# Figure S1. GSE244118 quality control

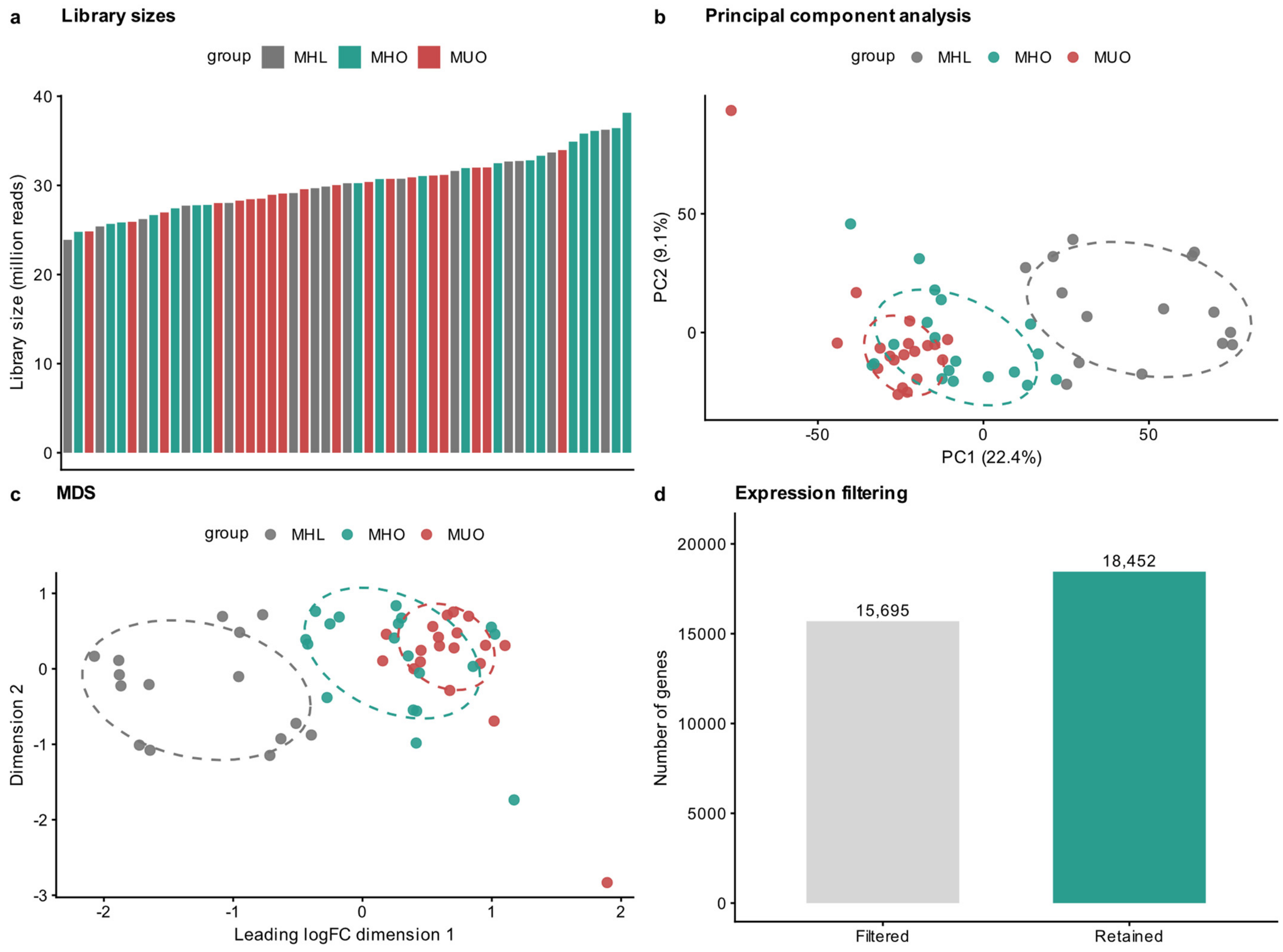


Library sizes, principal components, multidimensional scaling and expression filtering in the discovery dataset. The retained analysis comprises MHL (n = 15), MHO (n = 19) and MUO (n = 19). Expression-based QC is descriptive and is not evidence of independent phenotype validation.

# Figure S2. Discovery expression patterns

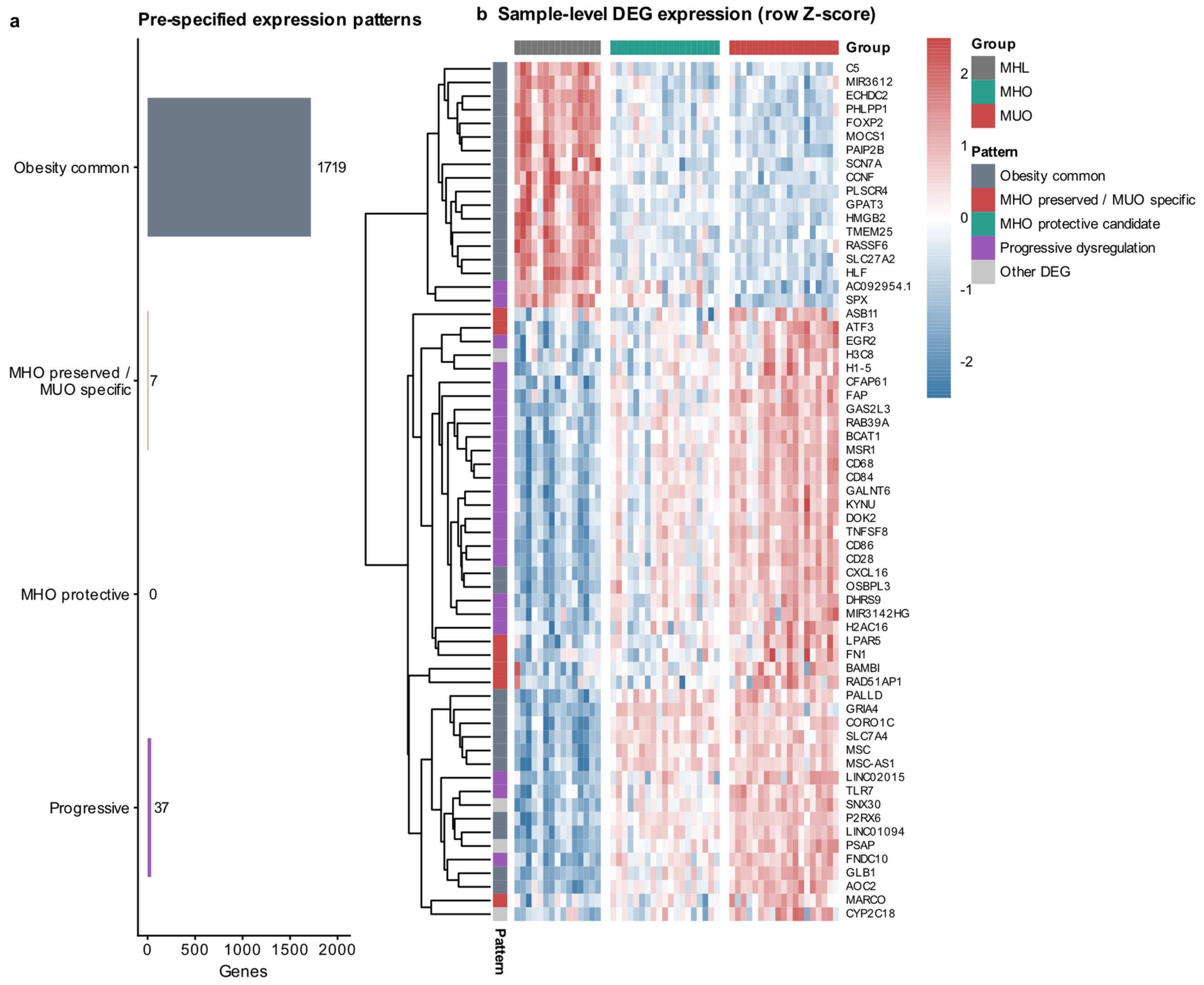


Counts of the original predefined expression-pattern categories and the associated selected-gene sample heatmap. Columns are grouped by phenotype. These categories are discovery-stage descriptions, not independently validated protective or causal mechanisms.

# Figure S3. Discovery group-mean expression

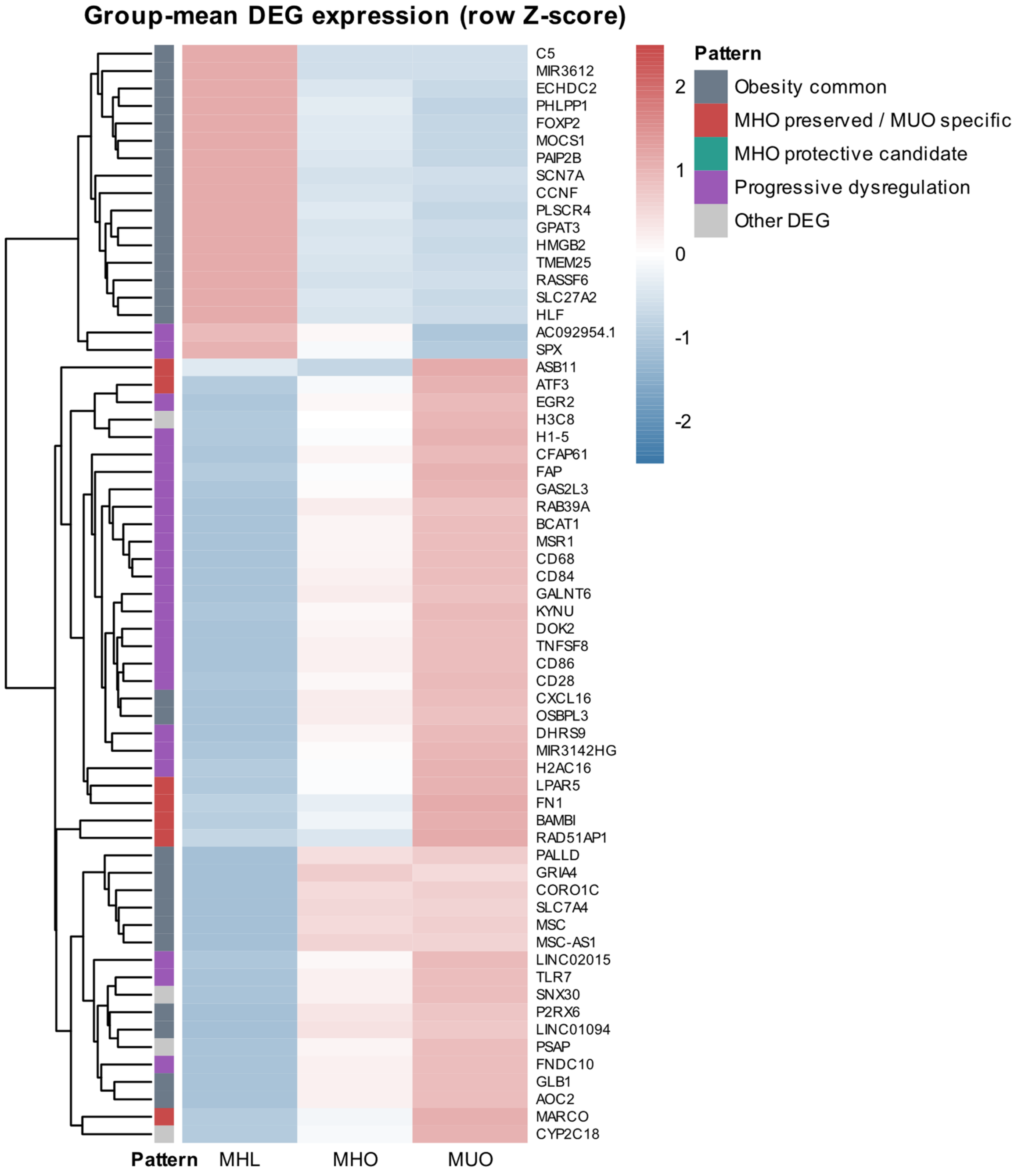


Group means for the selected discovery genes across MHL, MHO and MUO. Row-standardized expression emphasizes relative patterns; this selected-gene display is descriptive and is not a test of longitudinal progression.

# Figure S4. Exploratory discovery Reactome pathways

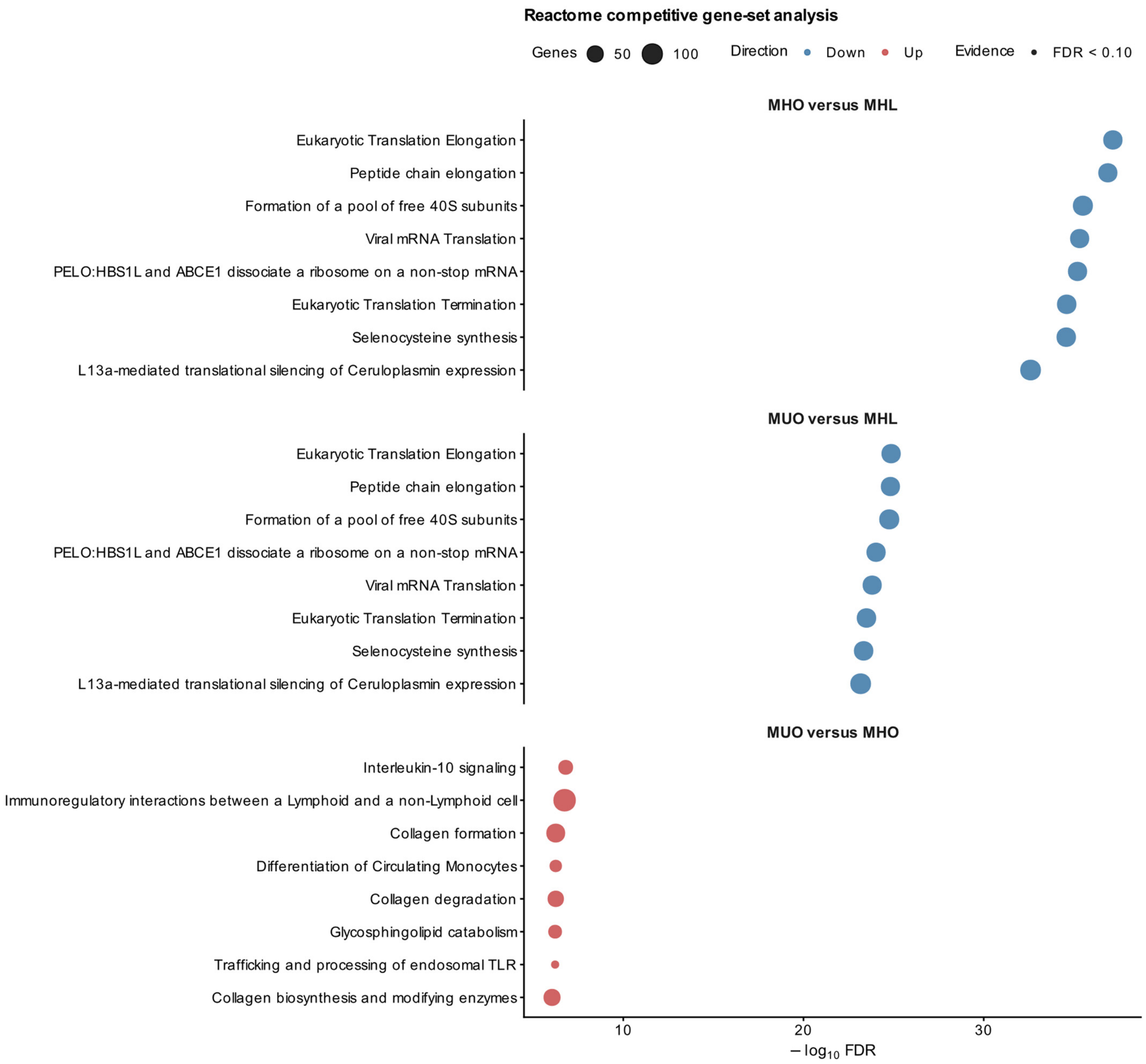


Selected CAMERA pathways for the three discovery contrasts. IMPORTANT: the historical panel uses FDR < 0.10 for exploratory pathway display, as shown in its legend. This must not be confused with the FDR < 0.05 criterion used for the formal cross-dataset pathway synthesis. Complete discovery pathway statistics are provided under full_results.

# Figure S5a. GSE152991 quality control

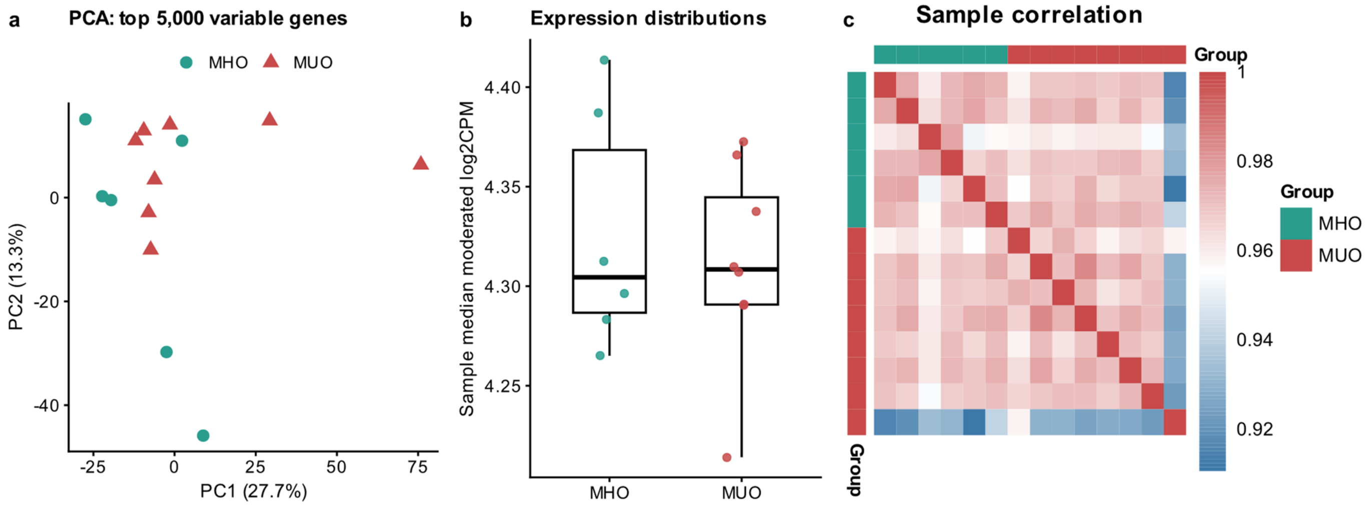


Principal components, sample median moderated log2CPM and sample correlations in the participant-nonoverlapping RNA-sequencing holdout (MHO n = 6; MUO n = 8). The processed matrix is author-supplied moderated log2CPM, not a raw count or FPKM matrix.

# Figure S5b. GSE152991 gene-level concordance

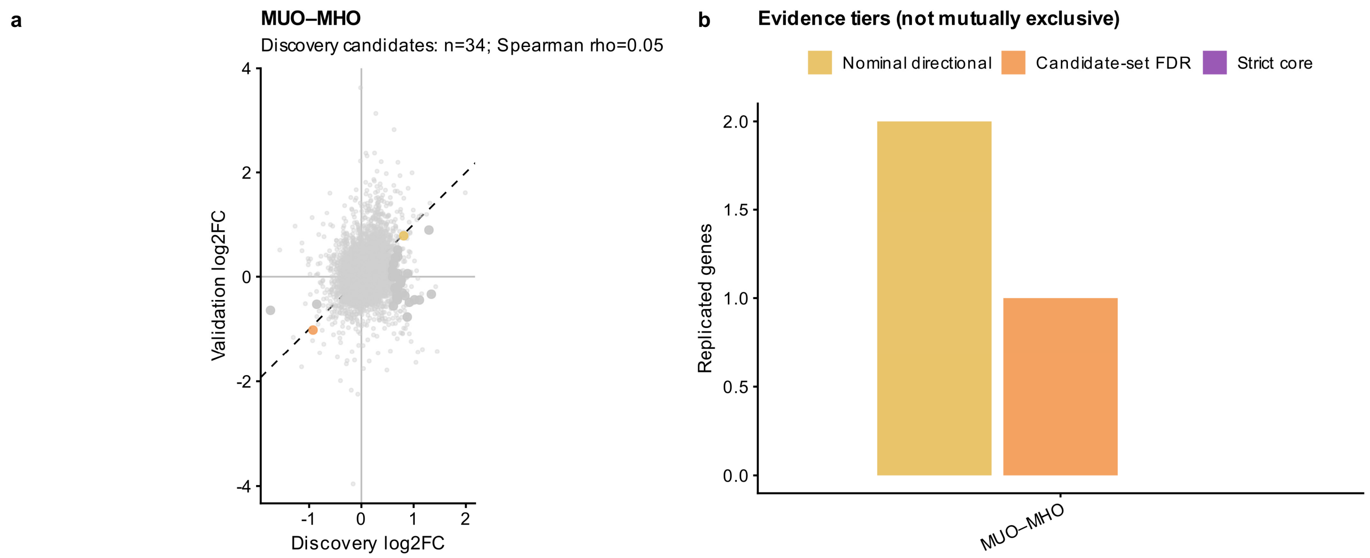


Discovery-versus-holdout effects for mapped discovery candidates and the original nominal, candidate-set and strict replication summaries. Only the 14 participant-nonoverlapping obese samples are used. Directional agreement alone is not a statistically validated gene signature.

# Figure S5c. Exploratory GSE152991 pathway concordance

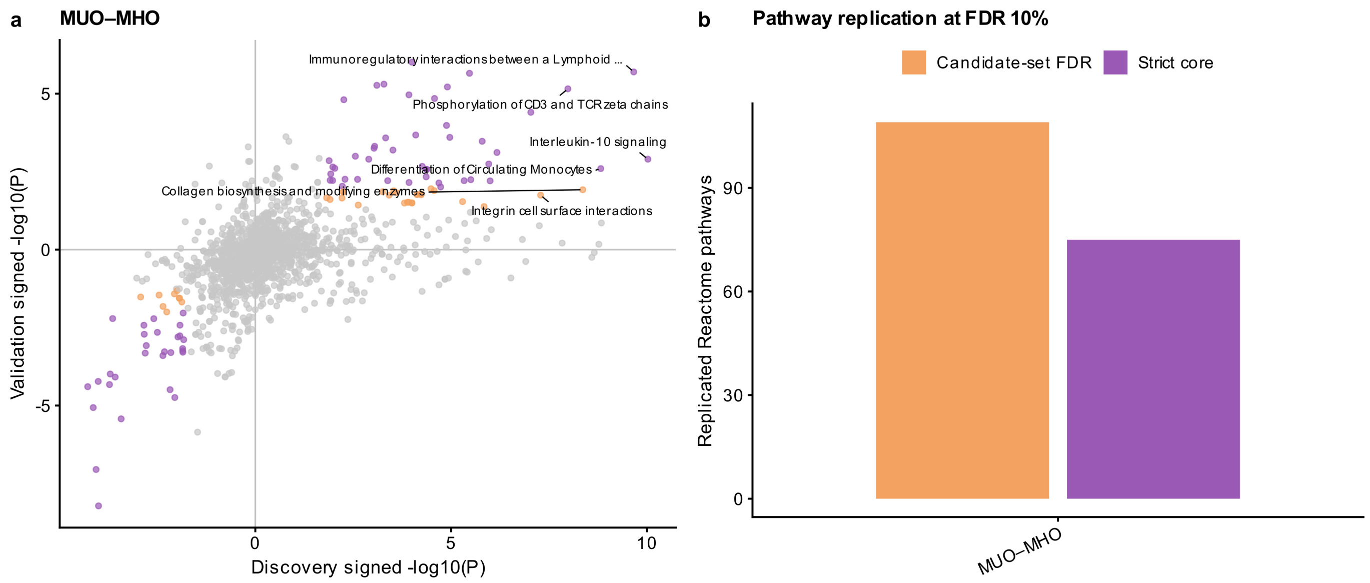


Signed pathway evidence and the original pathway-replication summaries. IMPORTANT: the replication panel is explicitly exploratory at FDR < 0.10. Table S14 also reports the stricter FDR < 0.05 summary; formal all-pathway meta-analysis results are in Table S21.

# Figure S6a. GSE55200 quality control

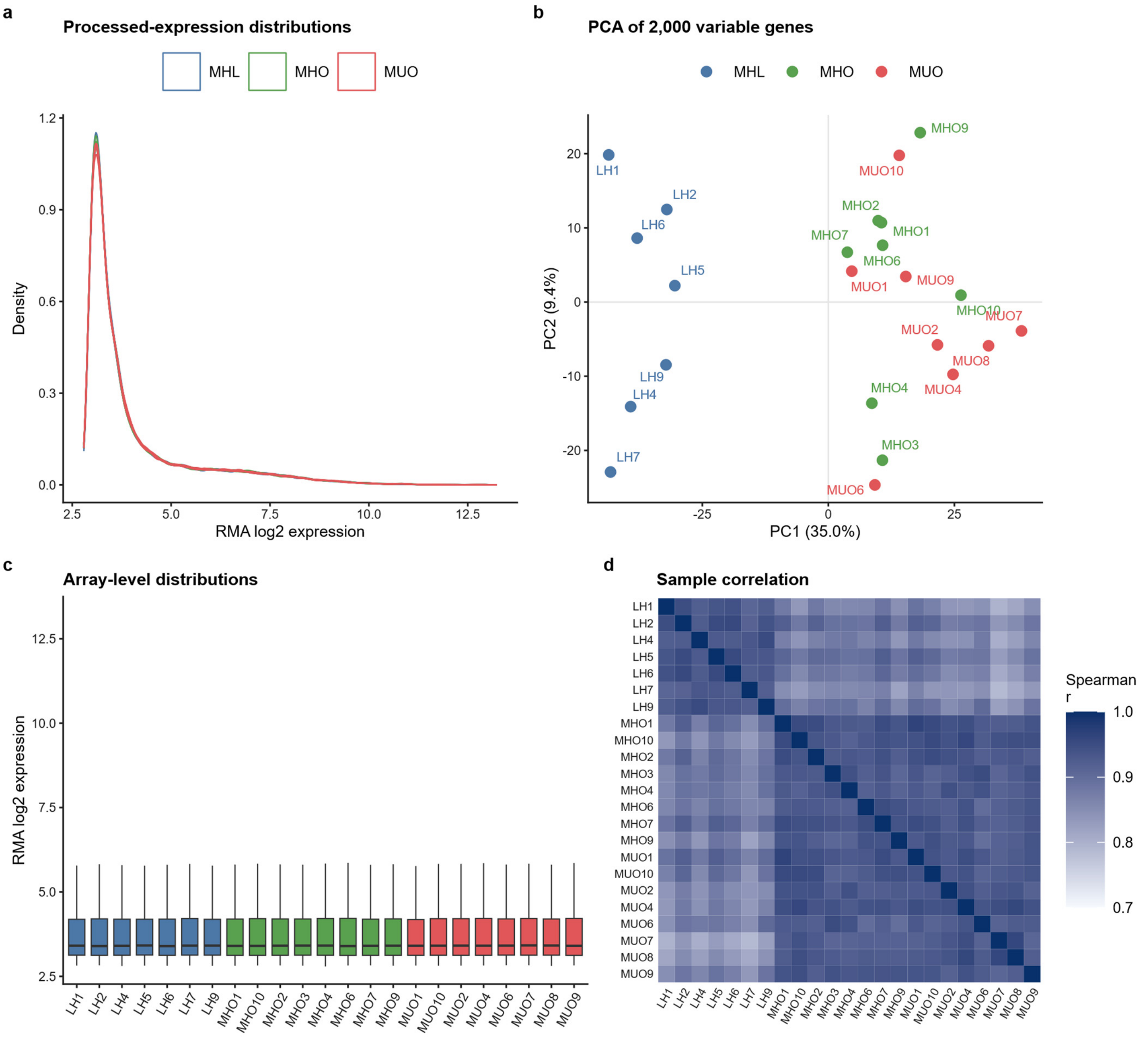


Expression density, principal components, sample boxplots and sample correlations in the cross-platform supportive microarray dataset (MHL n = 7; MHO n = 8; MUO n = 8). Groups were defined using anthropometric and metabolic criteria in the original publication (Badoud et al., 2014, doi:10.1021/pr500416v, p. 3456), not expression clustering. Each analyzed group contained two men (p. 3458).

# Figure S6b. GSE55200 candidate-gene support

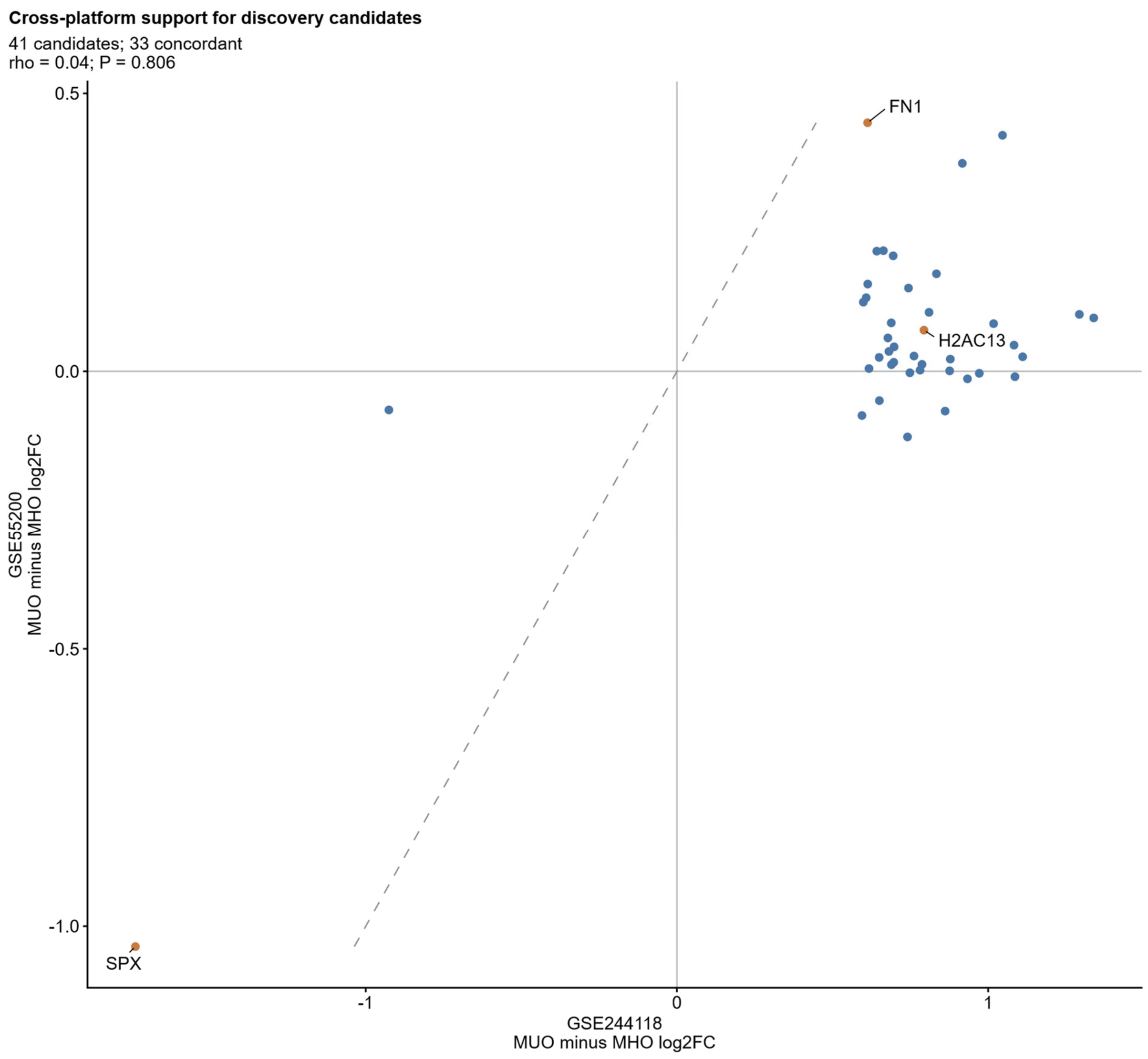


Discovery-versus-microarray effects for all 41 mapped candidate genes, of which 33 have concordant directions. Orange points and labels identify nominal directional support (SPX, H2AC13 and FN1); blue points denote the remaining candidates. None passed candidate-set FDR correction or strict genome-wide replication criteria. The dashed line indicates equal effects, with different axis scales. Spearman correlation across all 41 candidates was 0.040 (two-sided P = 0.806). The appropriate role is cross-platform supportive evidence, not definitive gene validation.

# Figure S6c. GSE55200 pathway support

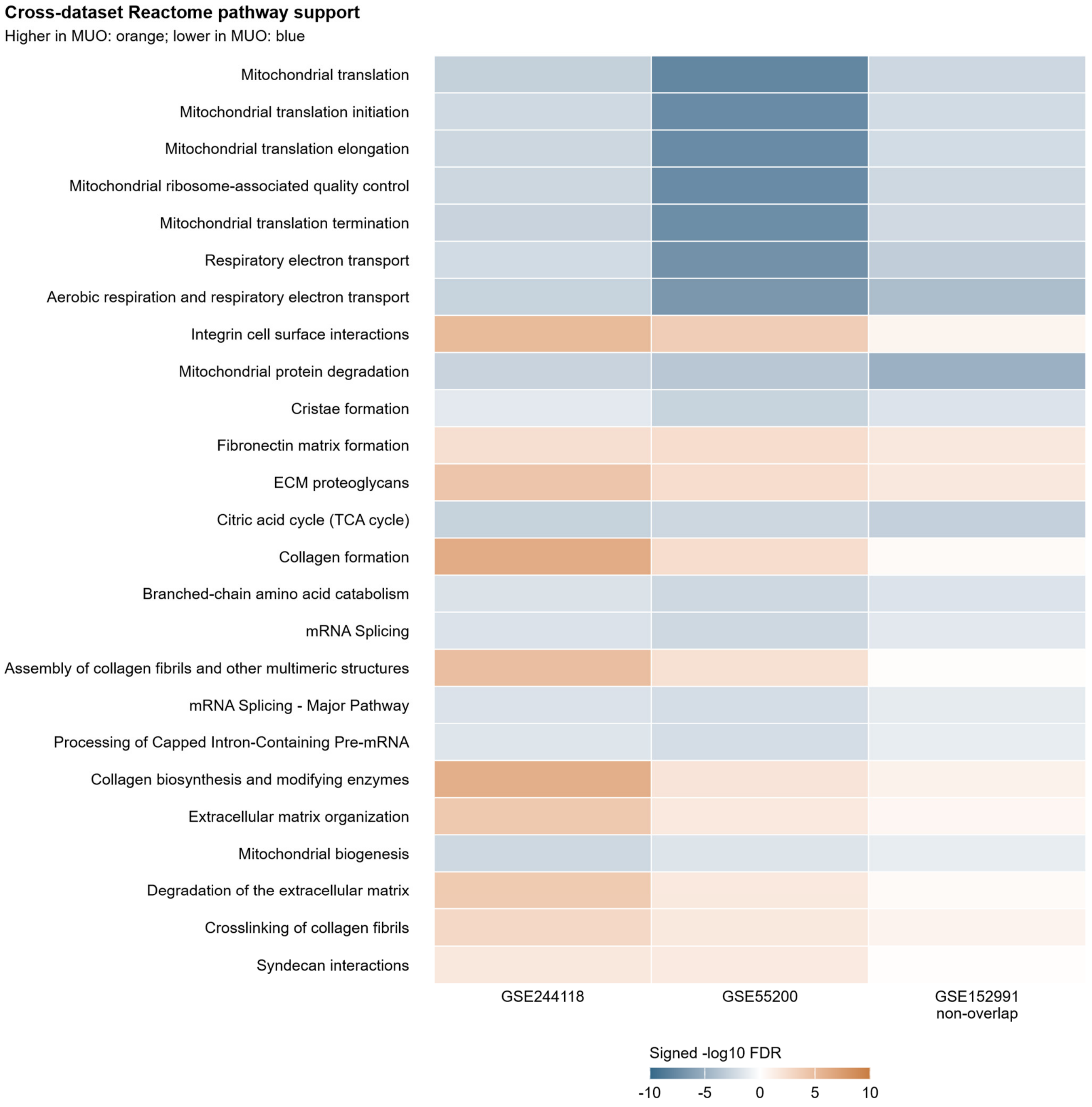


Selected Reactome pathway evidence across the three participant-distinct sample sets. GSE55200 provides cross-platform support, with differences in source-study metabolic-health definitions and platform limiting comparability. Pathway selection and overlapping Reactome gene membership preclude interpreting this panel as multiple independent mechanisms.

# Figure S6d. Exploratory GSE55200 pathway scores

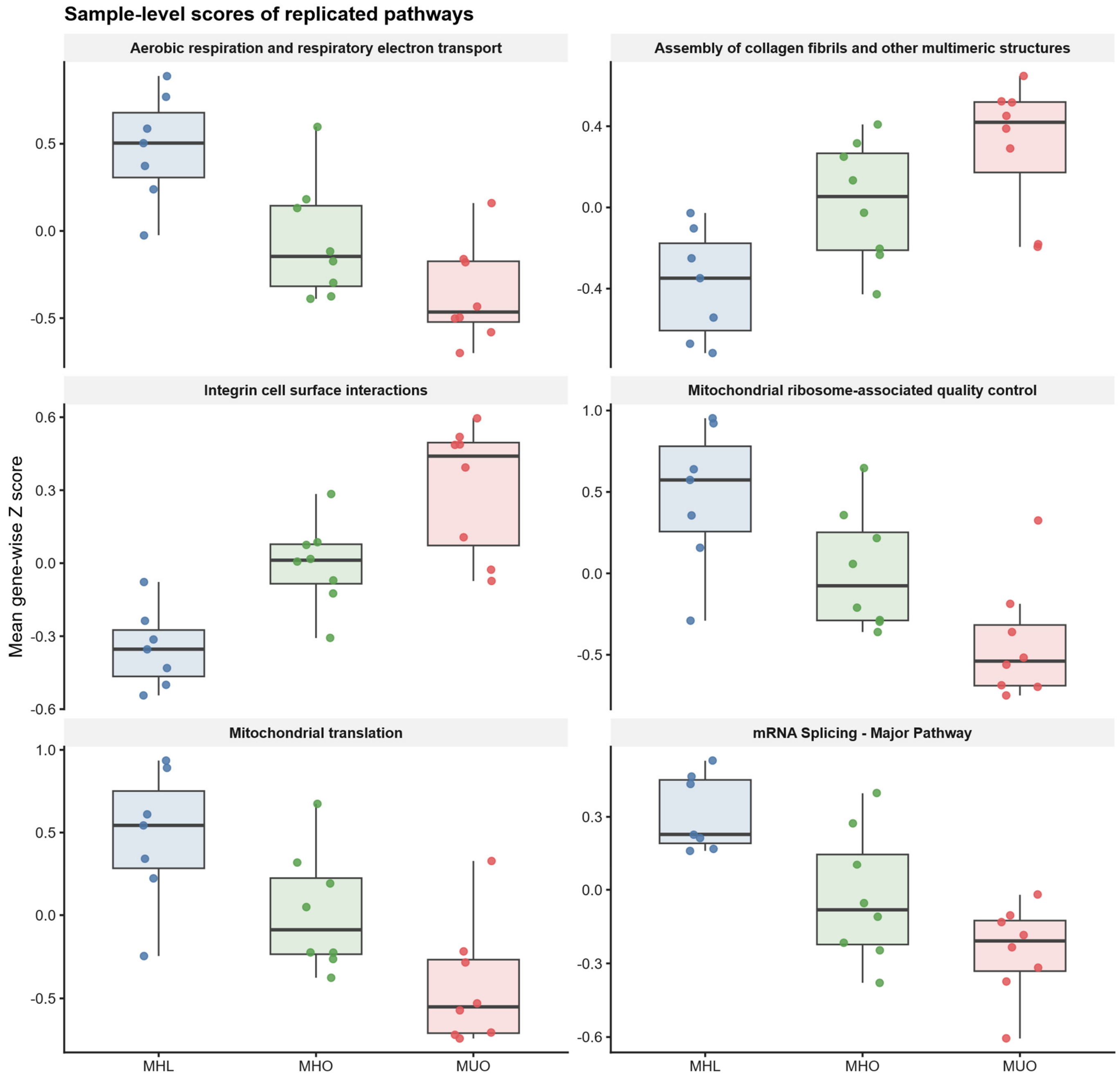


Sample-level scores for selected pathways across MHL, MHO and MUO. These historical pathway-specific gene-z-score summaries are exploratory and are not the final discovery-only 351-gene mitochondrial or 281-gene ECM modules. Final module membership and inferential tests are in Tables S2-S6.
